# Alkaline–Acid Intestine Environment Controlled by A Carbonic Anhydrase Gene Influences Synthesis of Sex Pheromone by Symbionts

**DOI:** 10.1002/advs.202511723

**Published:** 2025-09-14

**Authors:** Shiyu Gui, Lexin Xie, Zhenghao Wang, Yan Chen, Yi Xiao, Ziyun Lin, Jingxiang Chen, Yongyue Lu, Laurent Keller, Daifeng Cheng

**Affiliations:** ^1^ Department of Entomology South China Agricultural University Guangzhou 510640 China; ^2^ Social Evolut Unit Cornuit 8, BP 855, Ollon Chesieres CH‐1885 Switzerland

**Keywords:** carbonic anhydrase, rectal alkaline environment, rectal Bacillus, sex pheromone

## Abstract

The intricate interplay between animals and their intestinal microbes is pivotal in shaping various aspects of animal biology. However, the degree to which hosts can modulate the activity of their intestinal microbes, as well as the underlying molecular mechanisms, remains poorly elucidated. The production of sex pheromones by rectal *Bacillus* in male *Bactrocera dorsali*s is triggered by the alkaline intestine environment. An experimental increase in pH lead to more sex pheromone production, or vice versa. pH modulates the synthesis quantity of sex pheromone by exerting an impact on the activity of enzyme synthesizing sex pheromone in *Bacillus*. Transcriptome analysis shows that a highly expressed carbonic anhydrase (CAh) gene in *B. dorsali*s is associated with alkaline rectal environment. CAh inhibitor feeding and RNAi targeting the CAh gene lead to a shift from alkaline to acidic conditions within rectum and subsequent decreasing in sex pheromone synthesis and mating. This study provides novel insight into the influence of intestinal environment on intestinal microbes and has significant implication for understanding the molecular mechanism underlying sex pheromone synthesis by symbionts in insects.

## Graphical abstract

A model proposing that elevated rectal expression of CAh1 in males alkalinizes the environment to regulate Bacillus production of a male‐specific sex pheromone.

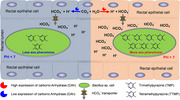

## 1 Introduction

The animal gut harbors a diverse and intricate microbial community that is essential for the overall health of the host.^[^ ^1^, ^2^, ^3^ ^]^ The complex interplay between animals and their beneficial microbes plays a pivotal role in shaping host biology.^[^ ^4^, ^5^, ^6^, ^7^, ^8^, ^9^, ^10^, ^11^, ^12^ ^]^ A few studies have even demonstrated the involvement of intestinal bacteria in mediating pheromone‐based communication among animals. Early research revealed that gut microbes in beetles and locusts can synthesize pheromones with significant impacts on host behavior.^[^ ^13^, ^14^, ^15^, ^16^ ^]^ Subsequent investigations have uncovered the ability of gut microbes to influence kin recognition,^[^ ^17^ ^]^ aggregation,^[^ ^18^ ^]^ development, and olfactory behavior^[^ ^19^ ^]^ in insect hosts through either direct production or modulation of pheromone synthesis.

In return, gut bacteria rely on their host to maintain a stable ecosystem and access nutrients.^[^ ^20^ ^]^ Consequently, the activity and survival of gut bacteria may be significantly impacted by the physical conditions within the host's gut.^[^ ^21^, ^22^, ^23^, ^24^ ^]^ The intestinal environment plays a crucial role in facilitating successful colonization by symbiotic bacteria and also influences bacterial function within the host.^[^ ^25^, ^26^, ^27^, ^28^, ^29^, ^30^ ^]^ The pH in the gut lumen is actively regulated and typically hovers around 7.^[^ ^31^ ^]^ However, lepidopteran larvae exhibit extreme alkalinity in their midguts, with pH levels reaching values up to 11–12,^[^ ^32^, ^33^ ^]^ resulting in the exclusion of most bacteria. In termites, pH ranges from 5 to > 12 within compartmentalized guts of certain species.^[^ ^26^ ^]^ The extreme alkalinity present in some compartments of termite guts supports the proliferation of specialized alkaline‐tolerant symbiotic bacteria.^[^ ^26^, ^34^ ^]^

The oriental fruit fly, *Bactrocera dorsalis*, is a significant pest insect causing extensive damage to agricultural and horticultural production,^[^ ^35^, ^36^, ^37^ ^]^ and harbors a diverse array of bacteria in its gut.^[^ ^38^ ^]^ Our previous research has manifested that the male‐specific sex pheromone (trimethylpyrazine‐TMP and tetramethylpyrazine‐TTMP) of *B. dorsalis* and *B. cucurbitae* is synthesized in the rectum, which is the ultimate portion of the hindgut, located close to the anus, by symbiotic *Bacillus* using glucose, amino acids and ammonium provided by the host as precursors.^[^ ^15^, ^39^, ^40^, ^41^, ^42^ ^]^ Although *Bacillus* is widely identified in numerous insect species,^[^ ^43^, ^44^, ^45^ ^]^ TMP and TTMP appear to be specific to *Bactrocera*, uncovering the particularity and complexity of the synthetic sex pheromone produced by rectal *Bacillus* in *Bactrocera*. Although studies have demonstrated that the environment within the insect gut can exert a significant influence on the composition and function of gut bacteria,^[^ ^25^, ^26^, ^27^, ^28^, ^29^, ^30^ ^]^ it remained unclear whether the environment in the gut of *Bactrocera* could regulate the synthesis of sex pheromones in the rectum by the *Bacillus*, which are crucial for courtship. This study utilized *B. dorsalis* as a model organism to investigate if hosts can regulate gut bacterial function and sex pheromone production by manipulating the gut environment. We demonstrated that the rectal pH of the male rectum could vary between acidic and alkaline with development and daily rhythm, which can affect the synthesis of sex pheromones by rectal bacteria. The study offers novel perspectives on the influence of the intestinal environment on intestinal microbes and has significant implications for understanding the molecular mechanisms underlying the synthesis of sex pheromones by symbionts in insects.

## 2 Results

### 2.1 Alkaline Rectal Environment is Positively Associated with Sex Pheromone Synthesis

**Figure 1:**
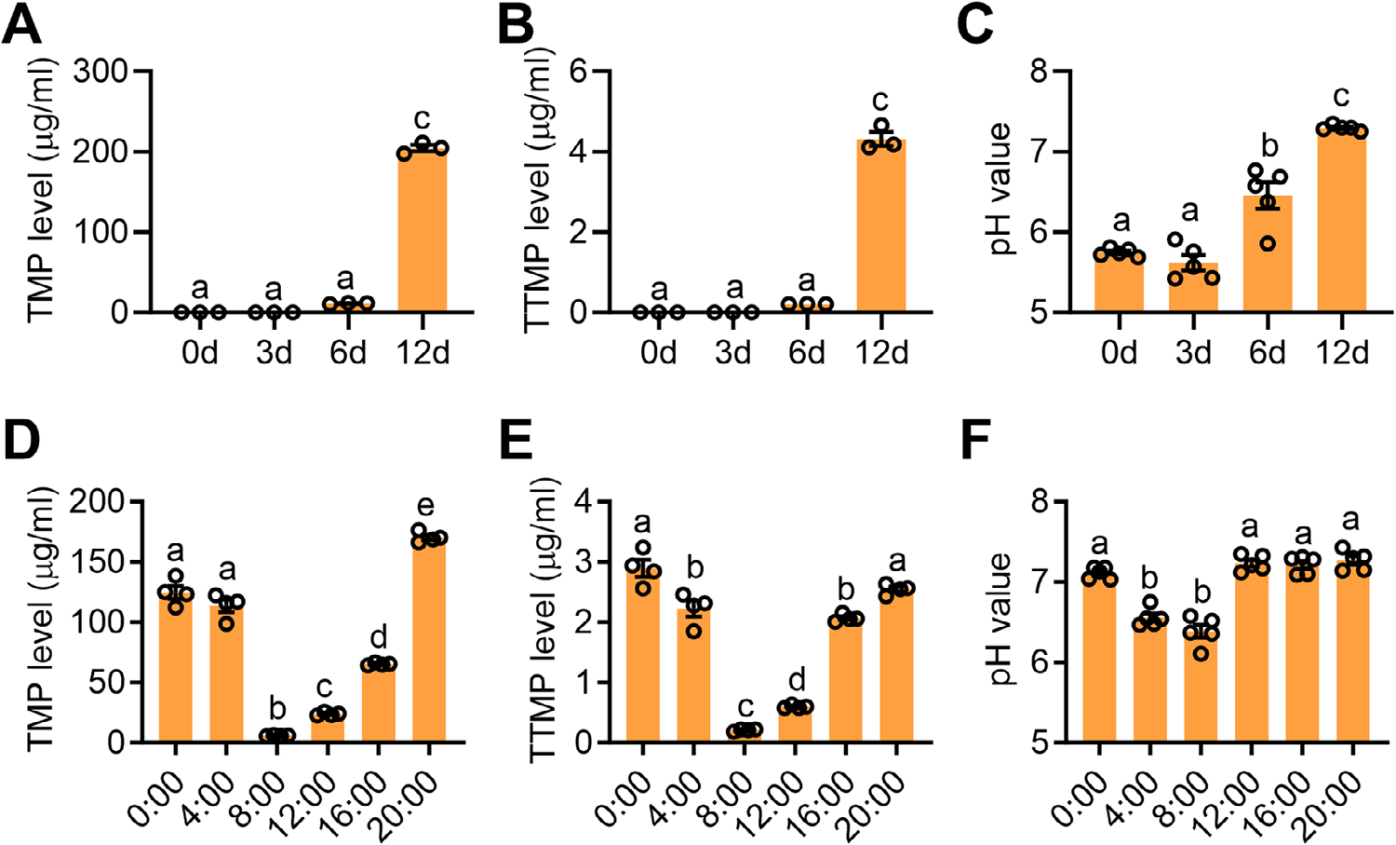
Sex pheromone synthesis was positively related to pH in the male rectum. A) TMP levels in the rectum of male at 20:00 across different developmental stages (*n* = 3, *F*_(3,8)_ = 2170, *p* < 0.0001). B) TTMP levels in the rectum of male at 20:00 across different developmental stages (*n* = 3, *F*_(3,8)_ = 573.4, *p* < 0.0001). C) pH in the rectum of male at 20:00 across different developmental stages (*n* = 5, *F*_(3,16)_ = 65.51, *p* < 0.0001). D) TMP levels in the male rectum at different times of day (*n* = 4, *F*_(5,18)_ = 393.2, *p* < 0.0001). E) TTMP levels in the male rectum at different times of day (*n* = 4, *F*_(5,18)_ = 178.8, *p* < 0.0001). F) pH in the male rectum at different times of day (*n* = 5, *F*_(5,24)_ = 50.73, *p* < 0.0001).

We initially investigated when sex pheromone will be produced in the rectum of males. The results showed that sex pheromone‐TMP and TTMP are exclusively synthesized in mature males (**Figure** **1**A,B; Figure S1A, Supporting Information). Given the importance of rectal *Bacillus* species for sex pheromone synthesis, we further compared rectal bacterial composition of males at different developmental stages. Though significant differences were observed in rectal bacterial composition between males at different stages (Permannova: *R* = 0.769, *P* = 0.001, Figure S1B,C and Dataset S1, Supporting Information), there was no significant disparity in *Bacillus* absolute abundance between males at different stages (Figure S1C,D, Supporting Information). Pearson correlation analysis further revealed no significant correlation between the levels of sex pheromone and the abundance of rectal *Bacillus* in males at different developmental stages (TMP vs *Bacillus* abundance: *R* = 0.23, *P* = 0.48 (Data passed normality test by D'Agostino & Pearson test, TMP :K2 = 3.842, *P* = 0.147; *Bacillus* abundance: K2 = 1.578, *P* = 0.454); TTMP vs *Bacillus* abundance: *R* = 0.21, *P* = 0.52 (Data passed normality test by D'Agostino & Pearson test, TTMP : K2 = 4.243, *P* = 0.12; *Bacillus* abundance: K2 = 1.578, *P* = 0.454), Figure S1E,F, Supporting Information). These results suggest that *Bacillus* abundance does not account for variations in sex pheromone synthesis in mature males. As pyrazine production by *Bacillus* is dependent on an alkaline environment,^[^ ^46^ ^]^ we hypothesized that the pH in the rectum may influence whether *Bacillus* synthesizes sex pheromone in males. pH detecting revealed an alkaline environment (pH 7.3) in the rectum of mature males, while an acidic environment (pH 5.6–6.4) was observed in young males (Figure 1C). Pearson correlation analysis demonstrated a significant positive correlation between sex pheromone levels and alkaline rectal pH in males of different developmental stages (TMP vs pH: *R* = 0.87, *P* = 0.002; TTMP vs pH: *R* = 0.86, *P* = 0.0003). On the other hand, we observed synthesis of sex pheromone in mature males peaked at 20:00 and reached a low point at 8: 00 (Figure 1D,E; Figure S2A, Supporting Information). Though significant differences were observed in rectal bacterial composition between males at different times of day (Permannova: *R* = 0.415, *P* = 0.001, Figure S2B,C and Dataset S2, Supporting Information), no significant higher absolute abundance for rectal *Bacillus* was detected in mature male rectums at 20:00 (Figure S2C,D, Supporting Information). Pearson correlation analysis further revealed no significant correlation between the levels of sex pheromone and the abundance of rectal *Bacillus* in males at different times of day (TMP vs *Bacillus* abundance: *R* = −0.22, *P* = 0.3 (Data passed normality test by D'Agostino & Pearson test, TMP : K2 = 5.053, *P* = 0.08; *Bacillus* abundance: K2 = 0.173, *P* = 0.917); TTMP vs *Bacillus* abundance: *R* = −0.21, *P* = 0.33 (Data passed normality test by D'Agostino & Pearson test, TTMP : K2 = 4.79, *P* = 0.09; *Bacillus* abundance: K2 = 0.173, *P* = 0.917), Figure S2E,F, Supporting Information). Noteworthy, alkaline pH levels (7.3) were detected in the mature male rectum at the time when sex pheromone levels peaked (Figure 1F). Pearson correlation analysis also revealed a significant positive correlation between sex pheromone levels and alkaline rectal pH in males at different times of day (TMP vs pH: *R* = 0.3988, *P* = 0.0356; TTMP vs pH: *R* = 0.4331, *P* = 0.0213). Collectively, these findings suggest that the synthesis of sex pheromone by rectal *Bacillus* may be influenced by the alkaline pH in the rectum.

Different letters above the error bars indicate significant differences at the 0.05 level analyzed by ANOVA followed by Tukey's test.

### 2.2 Alkaline pH Determinates Sex Pheromone Synthesis Activity of Rectal Bacteria

**Figure 2:**
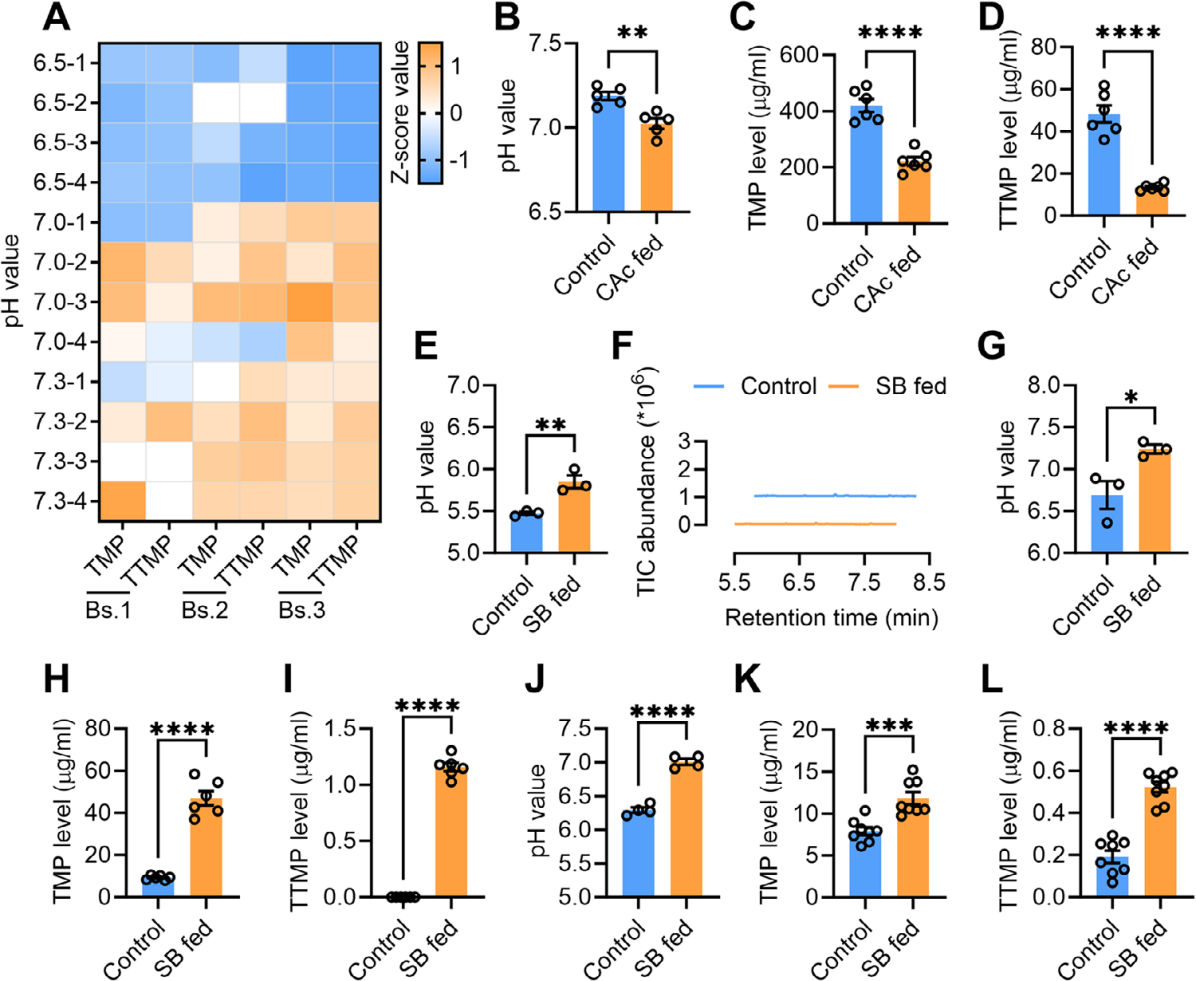
Influence of pH on the sex pheromone synthesis activity of rectal *Bacillus* species. A) Sex pheromones produced by rectal *Bacillus* in culture media with different pH values. Bs.1: *Bacillus pumilus*, Bs.2: *Bacillus altitudinis*, Bs.3: *Bacillus safensis*. Prior to heat mapping, the content of sex pheromone in each column was standardized by means of the Z‐score standardization method. B) Influence of CAc feeding on rectal pH in mature male (*n* = 5, *p* = 0.0033, independent sample Student's *t‐*test). C) Influence of CAc feeding on the TMP level in mature male (*n* = 6, *p* < 0.001, independent sample Student's *t‐*test). D) Influence of CAc feeding on the TTMP level in mature male (*n* = 6, *p* < 0.001, independent sample Student's *t‐*test). E) Influence of sodium bicarbonate (SB) feeding on rectal pH in 3‐day‐old male *(n* = 3, *p* = 0.0086, independent sample Student's *t‐*test). F) GC‐MS ion chromatograms of rectum extracts of 3‐day‐old male fed with sodium bicarbonate (SB). G) Influence of sodium bicarbonate (SB) feeding on rectal pH in 6‐day‐old male (*n* = 3, *p* = 0.0343, independent sample Student's *t‐*test). H) Influence of sodium bicarbonate (SB) feeding on the TMP level in 6‐day‐old male (*n* = 6, *p* < 0.0001, independent sample Student's *t‐*test). I) Influence of sodium bicarbonate (SB) feeding on the TTMP level in 6‐day‐old male *(n* = 6, *p* < 0.0001, independent sample Student's *t‐*test). J) Influence of sodium bicarbonate (SB) feeding on rectal pH in 12‐day‐old male at 8:00 (*n* = 4, *p* < 0.0001, independent sample Student's *t‐*test). K) Influence of sodium bicarbonate (SB) feeding on the TMP level in 12‐day‐old male at 8:00 (*n* = 8, *p* = 0.0005, independent sample Student's *t‐*test). L) Influence of sodium bicarbonate (SB) feeding on the TTMP level in 12‐day‐old male at 8:00 (*n* = 8, *p* < 0.0001, independent sample Student's *t‐*test).

To provide additional evidence that the pH in the rectum regulates sex pheromone synthesis by *Bacillus* species, we conducted in vivo and in vitro experiments to test the effect of different pH on sex pheromone synthesis. In a previous study, three pheromone producing *Bacillus* strains were isolated from the male rectum (15). Therefore, we initially assessed the capacity of these three strains (*Bacillus pumilus* (Bs.1), *Bacillus altitudinis* (Bs.2) and *Bacillus safensis* (Bs.3), respectively) to produce sex pheromone in media with varying pH levels (ranging from 6.5 to 7.3). We found that pH didn't affect the growth of *Bacillus* strains (Figure S3, Supporting Information) but significantly influenced sex pheromone produced; minimal pheromone were synthesized in acidic medium, whereas substantial amounts of sex pheromone were produced in alkaline medium (**Figure** **2**A; Figures S3,S4, Supporting Information). These results imply that although *Bacillus* is capable of synthesizing sex pheromone in an acidic medium, acid can significantly inhibit the quantity of sex pheromone synthesis (this should not be due to acid degradation of sex pheromone). Inducing decreased male rectal pH (7.0) through citric acid (CAc) feeding in mature male (12‐day‐old) resulted in significantly decreased levels of sex pheromone (Figure 2B–D; Figure S5A, Supporting Information). Consistently, mating ability of the CAc fed males decreased significantly (Figure S5B, Supporting Information). For 3‐day‐old males, feeding of sodium bicarbonate (SB) solution resulted in a significant increase in rectal pH (increasing from 5.47 to 5.85) (Figure 2E). However, the acidic rectal environment (pH 5.85) still caused the feeding of SB to fail to generate sex pheromone in 3‐day‐old males (Figure 2F). For 6‐day‐old males, feeding with SB solution led to a significant increase of rectal pH to an alkaline level (increasing from 6.69 to 7.24) (Figure 2G), accompanied by increased synthesis of sex pheromone (Figure 2H,I; Figure S5C, Supporting Information). Similarly, rectal pH shifted to alkaline (increasing from 6.293 to 7.008) in SB solution fed mature males at 8:00 (Figure 2J), consistently leading to enhanced synthesis of sex pheromone (Figure 2K,L; Figure S5D, Supporting Information). Together, these findings support the hypothesis that an alkaline pH environment within the male rectum is crucial for *Bacillus* species to produce sex pheromone.

In the figures, pH and sex pheromone level were measured at 20:00.

### 2.3 Alkaline pH is Vital for Synthesis of Sex Pheromone Catalyzed by Threonine Dehydrogenase (TDH) of *Bacillus*

**Figure 3:**
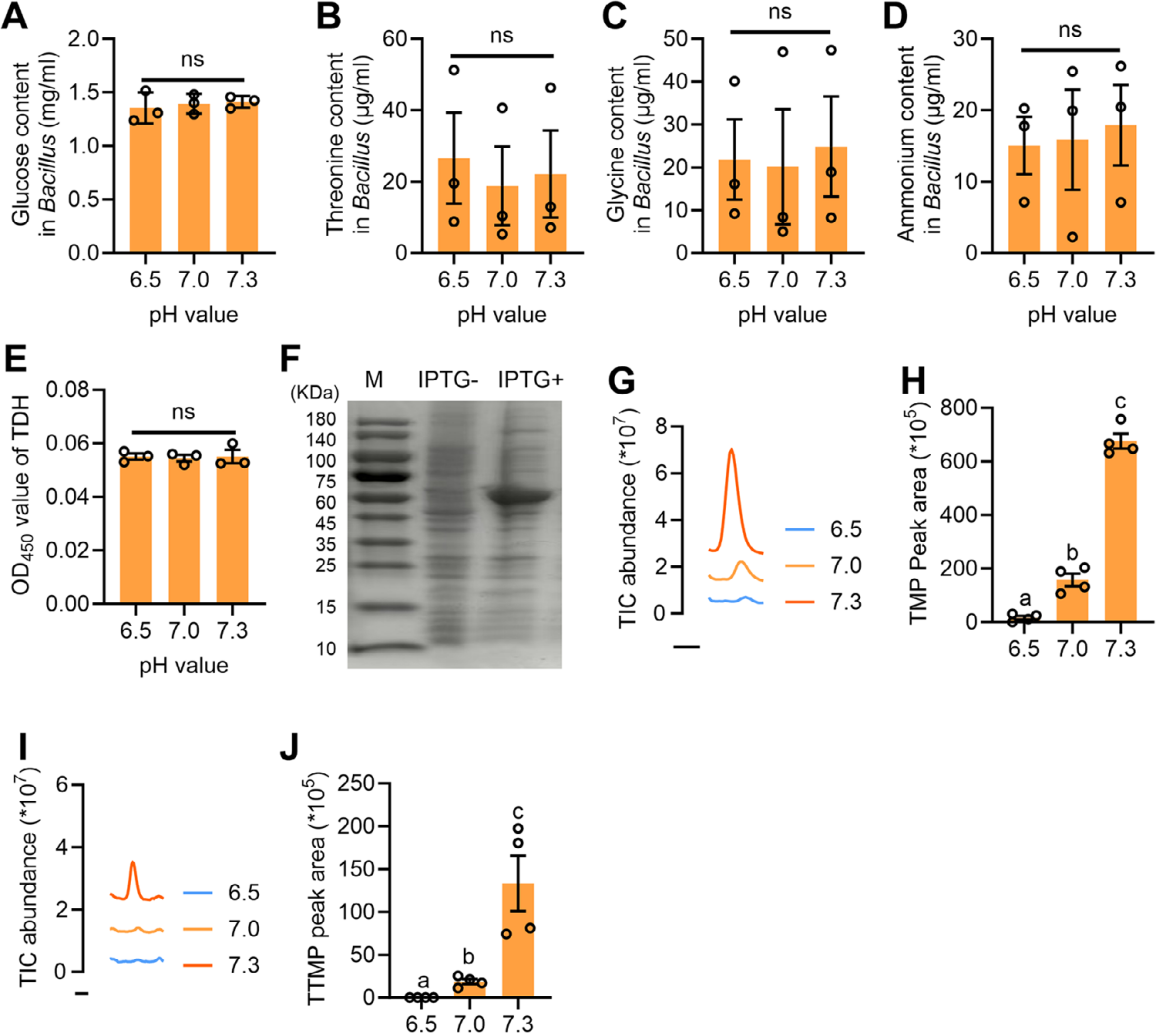
Alkaline pH controls sex pheromone synthesis by influencing the TDH activity. A) Glucose content in *Bacillus* cultived under different pH conditions (*n* = 3, *F*_(1.058, 2.116)_ = 0.9229, *p* = 0.4406, RM one‐way ANOVA). B) Threonine content in *Bacillus* cultived under different pH conditions (*n* = 3, *F*_(1.419, 2.838)_ = 11.07, *p* = 0.0548, RM one‐way ANOVA). C) Glycine content in *Bacillus* cultived under different pH conditions (*n* = 3, *F*_(1.325, 2.651)_ = 0.6329, *p* = 0.5361, RM one‐way ANOVA). D) Ammonium content in *Bacillus* cultived under different pH conditions (*n* = 3, *F*_(1.067, 2.134)_ = 0.9246, *p* = 0.4406, RM one‐way ANOVA). E) TDH content in *Bacillus* cultived under different pH conditions (*n* = 3, *F*_(2, 6)_ = 0.03792, *p* = 0.963, ANOVA). F) Electrophoretic profile of the TDH recombinant protein. G) GC‐MS ion chromatograms of TMP produced by incubating the TDH recombinant protein under various pH conditions. H) Contents of TMP produced by incubating the TDH recombinant protein under various pH conditions (*n* = 4, *F*_(2, 9)_ = 261.5, *p* < 0.0001, ANOVA). I) GC‐MS ion chromatograms of TTMP produced by incubating the TDH recombinant protein under various pH conditions. J) Contents of TTMP produced by incubating the TDH recombinant protein under various pH conditions (*n* = 4, Kruskal‐Wallis statistic = 10.2, *p* = 0.0002, Kruskal‐Wallis test ANOVA).

Previous studies have shown that *Bacillus* catalyzes the synthesis of TMP and TTMP from glucose, amino acids (threonine or glycine), and ammonium through its TDH and several spontaneous reactions.^[^ ^40^, ^46^ ^]^ To confirm the mechanism by which pH affects the synthesis of sex pheromone, we further measured the contents of glucose, amino acids (threonine or glycine), and ammonium in *Bacillus* strains cultured under different pH conditions. The results indicated that there were no significant differences in the contents of glucose, amino acids (threonine or glycine), and ammonium in *Bacillus* (**Figure** **3**A–D) suggesting that pH does not influence the efficiency of the precursor substances required for sex pheromone synthesis to enter the cells of *Bacillus*. Given that sex pheromone synthesis is also enzyme‐dependent, we further measured the TDH contents in the cells of *Bacillus* cultured under different pH conditions. Nevertheless, the results showed that pH had no effect on the TDH contents in the cells of *Bacillus* (Figure 3E). We further hypothesized that pH may affect the enzyme activity of TDH, thereby influencing sex pheromone synthesis. Consequently, we synthesized the recombinant TDH protein (Figure 3F) and evaluated the efficiency of this recombinant protein in catalyzing sex pheromone synthesis under various pH conditions. The results demonstrated that an alkaline pH significantly promoted the synthesis of sex pheromone by TDH. Conversely, an acidic pH remarkably hindered the synthesis of sex pheromone (Figure 3G–J). These results indicate that different pH values influence the synthesis quantity of sex pheromone by regulating the activity of the enzyme involved in sex pheromone synthesis.

Different letters above the error bars indicate significant differences at the 0.05 level. “ns” indicates no significant difference.

### 2.4 Alkaline pH‐Associated Carbonic Anhydrase (CAh) Gene Screening

**Figure 4:**
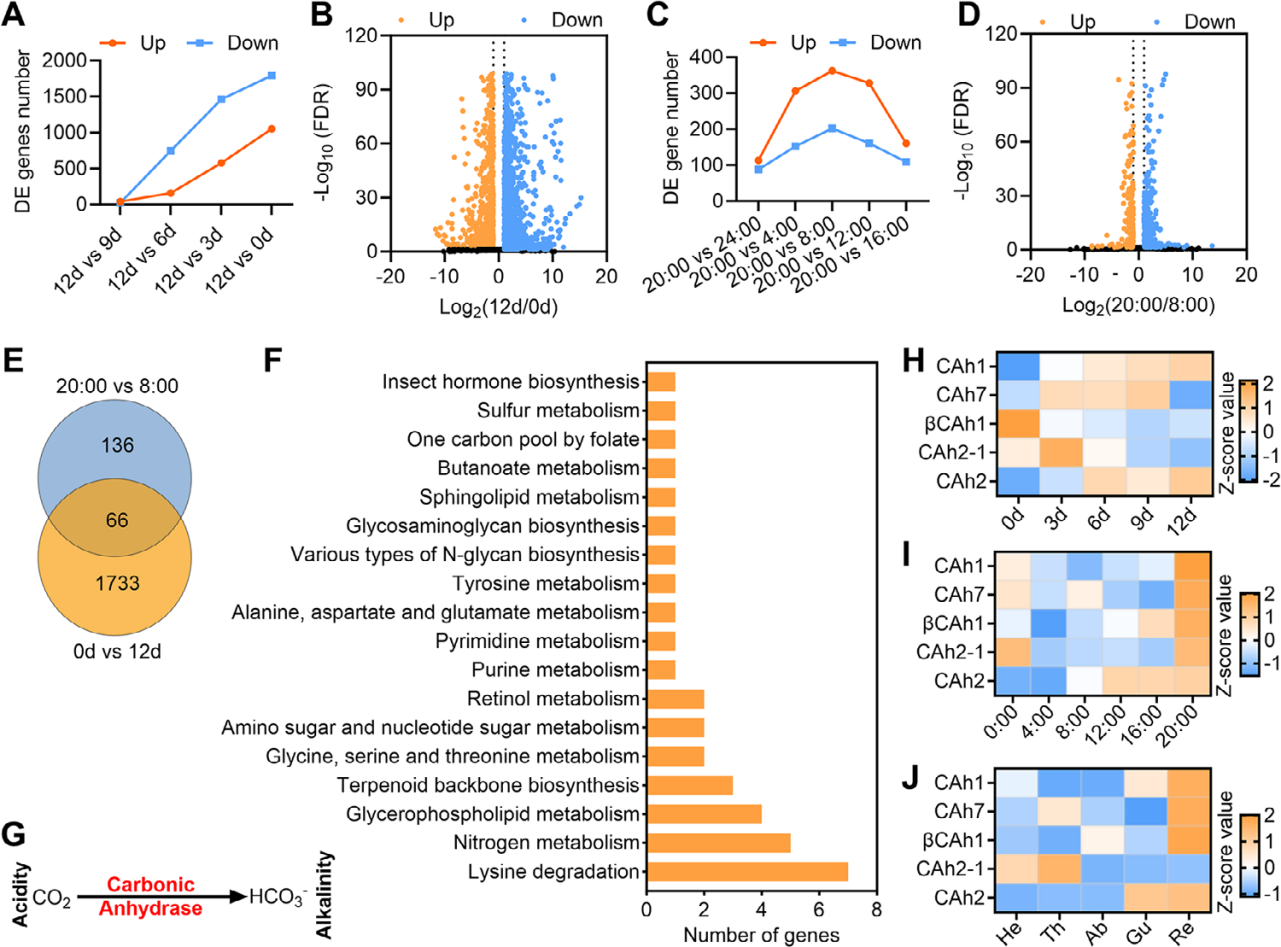
Alkaline rectum pH associated CAh genes screening. A) The number of differentially expressed genes in the rectum of 12‐day‐old males compared with younger males. B) DEGs between 12‐day‐old rectum and 0‐day‐old rectum. Up: Genes with higher expression in 12‐day‐old rectum; Down: Genes with lower expression in 12‐day‐old rectum. C) The number of differentially expressed genes in the rectum of males at 20:00 compared with males at other times of day. D) DEGs between rectum at 20:00 and 8:00. Up: Genes with higher expression in rectum at 20:00; Down: Genes with lower expression in rectum at 20:00. E) Screening of genes that were concurrently up‐regulated between 12 vs 0 d and 20:00 vs 8:00. F) KEGG enrichment analysis of the 66 concurrently up‐regulated genes in (E). G) Schematic diagram of CAh genes regulating the acidic–alkaline balance in organisms. H) Expression patterns of CAh genes in the rectum of males at 20:00 across different developmental stages. I) Expression patterns of CAh genes in the rectum of males at different times of day. J) Expression patterns of CAh genes in different tissues of mature males at 20:00. He: head, Th: thorax, Ab: abdomen, Gu: gut, Re: rectum.

Then, comparative rectal RNA‐seq analyses were conducted on males at various developmental stages and at different times of day to identify genes whose level of expression is associated with alkaline pH (Dataset S3, Supporting Information). For rectum at different development stages, more differentially expressed genes (DEGs) was identified as the age difference from 12‐day‐old males increased (**Figure** **4**A; Datasets S4–S6, Supporting Information), with 2855 DEGs (1799 up‐regulated and 1056 down‐regulated) being identified between 12 and 0 d (Figure 4B). Similarly, a greater difference in rectal sampling time for mature males led to the identification of a higher number of DEGs (Figure 4C; Datasets S7–S11, Supporting Information), with 564 DEGs (202 up‐regulated and 362 down‐regulated) being identified between 20:00 and 8:00 (Figure 4D). Screening results of the genes that were concurrently up‐regulated between 12 and 0 d as well as 20:00 and 8:00 indicated that there were 66 commonly up‐regulated genes (Figure 4E). As the pH variations in the rectum might be associated with the metabolism therein, we further carried out KEGG enrichment analysis with the 66 commonly up‐regulated genes. The results indicated that Lysine degradation, Nitrogen metabolism and Glycerophospholipid metabolism were the top three pathways enriched with the greatest number of up‐regulated genes (Figure 4F). It was notable that all five genes enriched in the nitrogen metabolism pathway were CAh genes. In the nitrogen metabolism pathway, it is assumed that carbonic anhydrase are involved in balancing the pH by catalysing the reversible hydration of carbon dioxide^[^ ^47^ ^]^ (Figure 4G). Previous studies also demonstrated that CAh knockdown significantly alters the pH of the *Drosophila* gut.^[^ ^48^, ^49^ ^]^ Therefore, we hypothesized that CAhs may influence the sex pheromone synthesis activity of rectal *Bacillus* by regulating the acidic‐alkaline balance in the male rectum. The expression profile of CAhs in the rectum across different development stages revealed that only CAh1 and CAh2 had higher expression levels in the 12‐day‐old male rectum (Figure 4H; Figure S6, Supporting Information). Moreover, it was found that the expression levels of all the screened CAh genes were significantly higher in the rectum of mature males at 20:00 than at other times (Figure 4I; Figure S7, Supporting Information). Tissue expression analysis also indicated that all CAh genes (except for CAh2‐1) were specifically expressed in the rectum of mature males (Figure 4J; Figure S8, Supporting Information). Collectively, these findings suggest that the rectum‐specifically expressed CAh genes, especially CAh1 and CAh2, might play a crucial role in regulating sex pheromone synthesis by maintaining an alkaline rectal pH.

In (A,B), samples for transcriptome sequencing were collected at 20:00. In (H–J), before heat mapping, the gene relative expression in each row was standardized using Z‐score standardization method.

### 2.5 CAh Enzyme Activity is Associated with Sex Pheromone Synthesis

**Figure 5:**
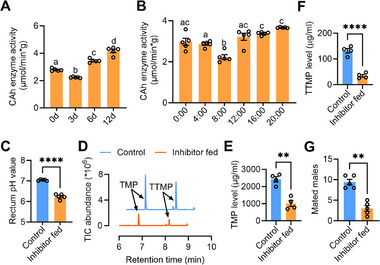
Higher CAh enzyme activities are associated with alkaline rectal pH and sex pheromone synthesis. A) Rectal CAh enzyme activity of male at 20:00 across different developmental stages (*n* = 5, *F*_(3,16)_ = 128.6, *p* < 0.0001, one‐way ANOVA). B) Rectal CAh enzyme activity of male at different time of a day (*n* = 5, *F*_(6,28)_ = 18.25, *p* < 0.0001, one‐way ANOVA). C) Influence of CAh enzyme inhibitor feeding on rectal pH (*n* = 5, *p* < 0.0001, independent sample Student's *t‐*test). D) GC–MS ion chromatograms of rectal extracts of males fed a CAh enzyme inhibitor. To prevent the peaks of sex pheromones from different treatments from overlapping one another, the chromatograms were shifted. E) Influence of CAh enzyme inhibitor feeding on the TMP level in mature male at 20:00 (*n* = 6, *p* = 0.0010, independent sample Student's *t‐*test). F) Influence of CAh enzyme inhibitor feeding on the TTMP level in mature male at 20:00 (*n* = 6, *p* < 0.0001, independent sample Student's *t‐*test). (G) Influence of CAh enzyme inhibitor feeding on male mating ability (*n* = 5, *p* = 0.0047, paired sample Student's *t‐*test).

To verify the impact of carbonic anhydrase on rectal pH and the synthesis of sex pheromone, we further investigated the relationship between rectal CAh enzyme activity and pH in male individuals at various developmental stages and times of day. The results demonstrated a significantly higher CAh enzyme activity in the rectum of mature males than younger males (**Figure** **5**A). Additionally, we also observed significantly higher CAh enzyme activity in the rectum of mature males at 20:00 compared to 8:00 (Figure 5B). Furthermore, administration of a CAh enzyme inhibitor could change the rectal alkaline environment to acidic in mature males at 20:00 (Figure 5C). And analysis of pheromone levels in the rectum of males exposed to inhibitors revealed a significant reduction in the concentrations of TMP and TTMP compared to control groups (Figure 5D–F). Consistently, mating ability of the CAh fed males decreased significantly (Figure 5G). These results indicate that carbonic anhydrase is responsible for the alkaline rectal environment for sex pheromone synthesis.

In (C–G), pH, sex pheromone, and mating behavior were measured at 20:00. Different letters above the error bars indicate significant differences at the 0.05 level analysed by ANOVA followed by Tukey's test.

### 2.6 CAh1 Controls Alkaline Rectal pH and Sex Pheromone Synthesis

**Figure 6:**
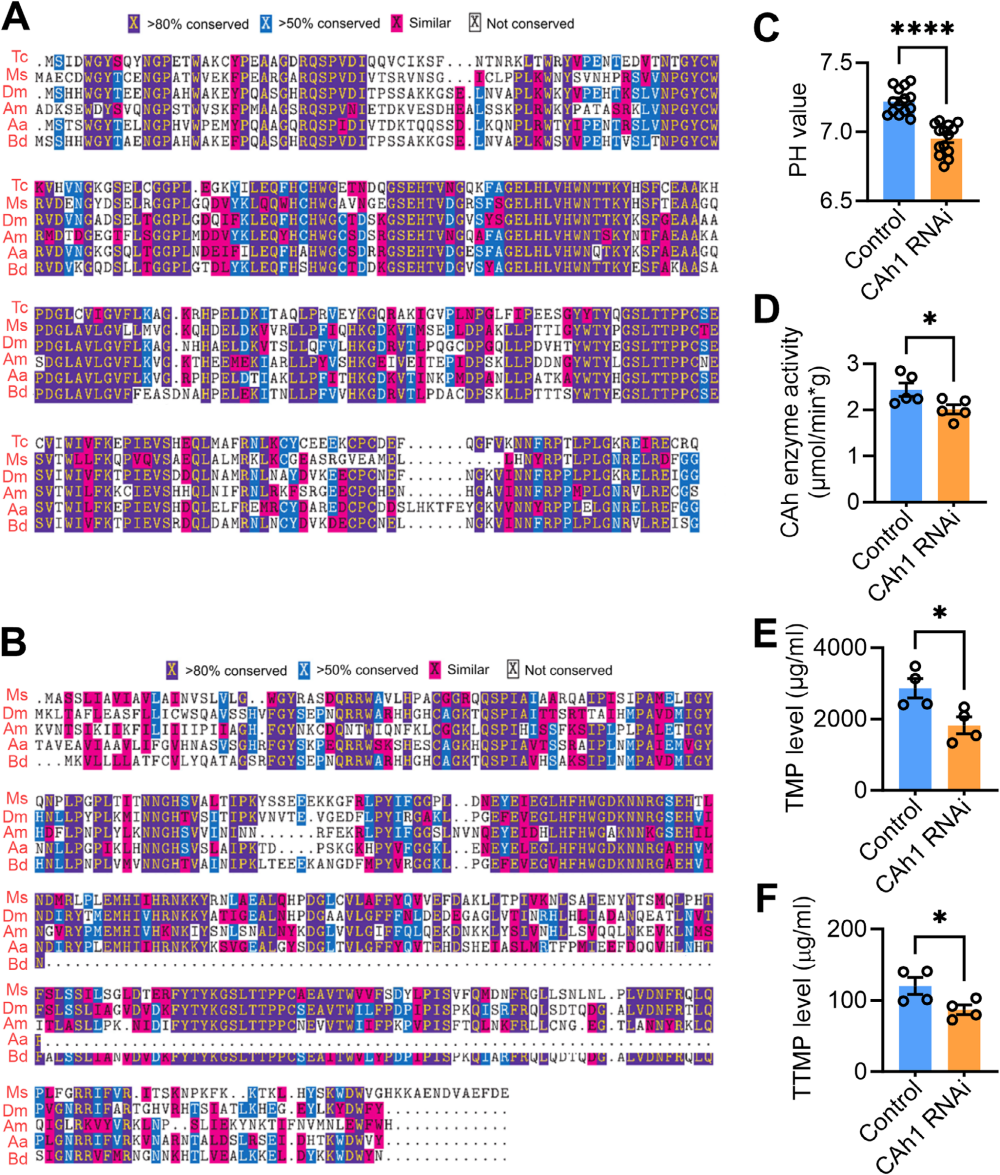
CAh1 contributes to alkaline rectal pH and sex pheromone synthesis. A) The similarity of the CAh1 amino acid sequence to that of other insect species. B) The similarity of the CAh2 amino acid sequence to that of other insect species. Tc: *Tribolium castaneum*; Ms: *Manduca sexta*; Dm: *Drosophila melanogaster*; Am: *Apis mellifera*; Aa: *Aedes aegypti*; Bd: *Bactrocera dorsalis*. C) Comparison of rectal CAh enzyme activity between males subjected to CAh1 knockdown and control males (*n* = 5, *p* = 0.0395, independent sample Student's *t‐*test). D) Influence of CAh1 knockdown on rectal pH (*n* = 5, *p* = 0.004, independent sample Student's *t‐*test). E) Influence of CAh1 knocking down on rectum TMP level (*n* = 4, *p* = 0.0265, Independent sample student's *t‐*test). F) Influence of CAh1 knocking down on rectum TTMP level (*n* = 4, *p* = 0.0479, Independent sample student's *t‐*test).

Given the reported involvement of CAh genes in regulating pH in fruit fly (*Drosophila melanogaster*) and mosquito (*Aedes aegypti*),^[^ ^50^, ^51^ ^]^ we conducted a comparative analysis to assess the similarity between CAh genes in *B. dorsalis* and those in other insects, including *D. melanogaster* and *A. aegypti*. A maximum likelihood phylogenetic tree, constructed using amino acid sequence alignments of CAh genes, revealed that the CAh genes of *B. dorsalis* were closely related to the known CAhs of other insects, with the exception of CAh2‐1 (Figure S9A, Supporting Information). Specifically, the amino acid sequences of CAh1 showed relative high resemblance to those identified in *D. melanogaster* and *A. aegypti*, while CAh2 had more different amino acid sites (Some sites are even absent) (**Figure** **6**A,B). Subsequently, we conducted RNAi experiments to investigate the associations between rectal CAh gene expression and pH. The results demonstrated that only down regulation of rectal CAh1 expression (Figure S9B, Supporting Information) led to a significant decrease in CAh enzyme activity and pH levels (acidic) (decreasing from 7.219 to 6.951) in male rectum (Figure 6C,D). And analysis of pheromone levels revealed a significant reduction in the concentrations of TMP and TTMP in CAh1 knocked down males compared to control groups (Figure 6E,F; Figure S9C, Supporting Information). Consistently, mating ability of the CAh1 knocked down males decreased significantly (Figure S9D, Supporting Information). However, decreased expression of any of the *CAh7, βCAh1*, *CAh2‐1*, and *CAh2* genes did not result in decreased rectal pH and mating ability in males (Figure S10, Supporting Information). These findings indicate that *CAh1* plays a pivotal role in sex pheromone synthesis by controlling the alkaline pH (about 7.3) in the rectum.

In (C–F), pH, sex pheromone, and enzyme activity were measured at 20:00.

## 3 Discussion

**Figure 7:**
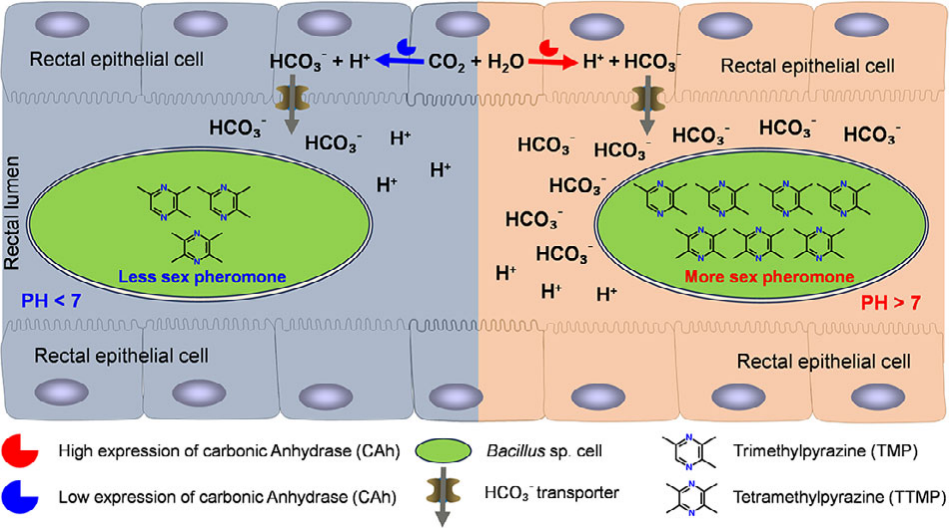
Model of rectal CAh‐mediated promotion of sex pheromone synthesis by rectal *Bacillus* species.

In this study, we have offered insights into the mechanism governing sex pheromone production by intestinal microbes in male *B. dorsalis*. We propose a model wherein males have evolved to express higher levels of the gene *CAh1* in the rectum to induce an alkaline environment (pH 7.3) and the production of a male‐specific sex pheromone by *Bacillus* (Figure 7). It is likely that in the rectal epithelium, the *CAh1* catalyzes CO_2_ to produce HCO_3_^−^ and H^+^. Additionally, transporters transfer HCO_3_^−^ into the rectum, making the alkaline rectal environment suitable for the sex pheromone synthesized by *Bacillus*. To the best of our knowledge, this is the first instance of a system in which an insect host can actively regulate intestinal microbe function by modulating fluctuations in intestinal pH **Figure** **7**.

The synthetic pathways of many chemicals by bacteria are known to be pH‐dependent^[^ ^46^, ^52^, ^53^, ^54^ ^]^ studies have demonstrated that pH can exert an influence on multiple biological processes of bacteria, such as the formation of bacterial biofilms and metabolic transitions, by being involved in the rearrangement of components within the intracellular transcriptome, proteome, and metabolome. Our study demonstrates that this is also applicable to intestinal microbes. Various biotic and abiotic factors, such as gut pH levels, oxygen availability, redox conditions, host dietary patterns or autoimmunity, can also impact the functions and community composition of gut bacteria in insects.^[^ ^31^, ^55^, ^56^, ^57^ ^]^ However, the mechanisms through which insect hosts influence microbial physiological activities have been largely overlooked. Given the specific advantages provided by certain microorganisms to their hosts, it is plausible that hosts have evolved diverse regulatory mechanisms over hundreds of millions of years to maintain the stability of particular microbial communities and regulate microbial activities. In order to ensure efficient resource utilization, host organisms may evolve regulatory mechanisms for the activities or functions of their particular symbiotic partners. Though the precise mechanisms through which alkaline and acidic conditions regulate sex pheromone synthesis remains unknown, our study is the first example demonstrating that intestinal *CAh* gene regulates pH levels, which subsequently impacts the function of intestinal microbes. Considering the active involvement of intestinal microbes in metabolic processes across vertebrate and invertebrate animals, further investigation is warranted to confirm whether such a mechanism is evolutionarily conserved.

In comparison to the foregut and midgut, the role of the insect rectum in food digestion and absorption is relatively minor.^[^ ^58^, ^59^ ^]^ However, it often exhibits specialization to facilitate adaptation to unfavorable environments.^[^ ^60^, ^61^, ^62^ ^]^ From a reproductive standpoint, a specialized rectum that stores *Bacillus* producing sex pheromone and regulates pH for pheromone synthesis and release may be more crucial than its digestive and absorptive roles in *B. dorsalis*. Owing to their smaller size and higher reproduction rate, bacteria possess considerably strong capabilities in chemical synthesis and decomposition^[^ ^63^ ^]^ This may allow insects to depend on bacteria to establish stable symbiotic relationships for the efficient synthesis of sex pheromone. On the contrary, insects may have developed sophisticated mechanisms to regulate the activity of gut bacteria in order to optimize their function. In addition, precisely controlling the pH levels in the intestines is undoubtedly one of the effective methods for regulating bacterial activity.

However, this study has some limitations for its methodology. How precisely the production of TMP/TTMP is regulated by pH in gut, *Bacillus* remains to be clarified. Although acidic conditions did not influence the growth of *Bacillus* (Figure S3, Supporting Information), and no biofilms or endospores were observed in the culture, the regulation at the level of microbial cell activity or protein activity on sex pheromone synthesis still needs to be elucidated in the future. Although the *CAh1* gene is highly expressed in the rectum of 6‐day‐old males, a discrepancy exists in the levels of pH, TMP and TTMP. Since *Bacillus* necessitates the utilization of glucose, amino acids, and ammonium provided by the host as precursors for the synthesis of TMP and TTMP,^[^ ^15^, ^39^, ^40^, ^41^, ^42^ ^]^ this indicates that the contents of the precursors might also have an impact on TMP and TTMP synthesis. Moreover, other factors (such as ammonium) that may affect rectal pH could also exist and have an influence on the synthesis of TMP and TTMP. While RNAi targeting other *CAh* genes did not decrease pH levels, RNAi targeting *CAh1* did not reduce pH to the same level as observed in young males and males fed with a *CAh* inhibitor. This suggests that other factors such as bacterial *CAh* genes, efficiency of RNAi on *CAh1*, and delays from gene expression to enzyme translation may also impact rectal pH levels. While we successfully utilized RNAi and inhibitors to decrease mRNA and enzyme activity of *CAh*, our ability to investigate the impact of gene overexpression on rectal pH was impeded by the absence of an in vivo overexpression system (e.g., GAL4/UAS system) for this non‐model species. This limitation resulted in our inability to induce younger male individuals to produce sex pheromone through overexpressing the CA1 gene.

## 4 Experimental Section

### Insect Preparation

The *B. dorsalis* strain used herein was collected from a carambola (*Averrhoa carambola*) orchard, which was open to the public for free, in Guangzhou, Guangdong Province, and reared under laboratory conditions (27 ± 1 °C, 12:12 h light:dark cycle (20:00 light off and 8:00 light on), 70–80% relative humidity (RH)). In subsequent studies, all sampling and observation times were in Beijing Time. A maize‐based artificial diet containing 150 g of corn flour, 150 g of banana, 0.6 g of sodium benzoate, 30 g of yeast, 30 g of sucrose, 30 g of paper towel, 1.2 mL of hydrochloric acid and 300 mL of water was used to feed the larvae. Adults were fed a diet consisting of a 1:1 ratio of yeast extract (protein food): sugar in a 35 × 35 × 35 cm wooden cage supplied with sterile water every day. The adult males and females were raised separately after emergence.

To measure the sex pheromone level and pH in rectum of male at different development stages, rectums were dissected and collected from males at 0, 3, 6, and 12 days after emergence (at 20:00), respectively. Then, the collected rectums were treated with the methods below for sex pheromone identification (60 rectums in one sample) and pH measurement (10 rectums in one sample). Similarly, rectums of sexually mature males (12 days after emergence) were collected at 0:00, 4:00, 8:00, 12:00, 16:00, and 20:00 during the day to measure sex pheromone level and pH at different time of day.

To verify whether sex pheromone production was controlled by rectal pH, males were fed CAc or sodium bicarbonate (SB). Briefly, newly emerged adults were fed sterile water containing citric acid (10 mg mL^−1^) or sodium bicarbonate (1 mg mL^−1^) in addition to a normal diet. Normally reared males were used as controls. Twelve days later, the rectal pH and sex pheromone level were compared (at 20:00).

To verify the effect of CA enzymatic activity on rectal pH and sex pheromones, mature males fed acetazolamide (a CA enzyme inhibitor) were used. Briefly, mature males (12‐days‐old) were fed sterile water containing acetazolamide (10 mg mL^−1^) in addition to a normal diet. Normally reared males were used as controls. 24 h later, the rectal pH, sex pheromone level, and mating ability of the males were compared (at 20:00).

### Mating Competition

Mating competition assays were performed in a 35 × 35 × 35 cm wooden cage according to a previous study.^[^ ^39^ ^]^ Briefly, 30 males in control group and 30 males in treatment group were colored with two different colors on the pronota and then were placed in one cage with 30 mature unmated females at 20:00. In this manner, the 60 males within the control group and the treatment group would contended to mate with the 30 females in the cage. Then, mating behavior of the flies was continuously observed visually for 2 h, and the number of males that mated in control and treatment group was recorded and compared. Successful mating was regarded as having taken place when the male's genitalia entered the female's body and remained therein for more than five minutes.

### Sex Pheromone Identification

Rectums of sixty males were dissected as one sample and extracted with 500 µL of n‐hexane by shaking (180 rpm) in a 16 °C incubator for 24 h. Then, GC–MS with an Agilent 7890B Series GC system coupled to a quadrupole‐type mass‐selective detector (Agilent 5977B; transfer line temperature: 230 °C, source temperature: 230 °C, ionization potential: 70 eV) was used to identify sex pheromones in the rectal extract. Helium at a constant pressure of 110.9 kPa was used as the carrier gas. One microlitre of each sample was automatically injected into the injector port (240 °C). The column temperature was maintained at 50 °C for 1 min, increased to 150 °C at 10 °C min^−1^, increased to 250 °C at 25 °C min^−1^ and maintained at 250 °C for 10 min. The mass spectra of the chemicals were compared with those listed in the NIST mass spectral library. Then, the identification of TMP and TTMP was confirmed by comparing their retention times and mass spectra with those of authentic standards. To determine the levels of TMP and TTMP in extracts, a standard curve was generated with authentic standards. The standard curves were prepared in triplicate (*n* = 3) using 4000, 2000, 1000, 500, 50, 5 and 0.5 µg mL^−1^ solutions of TMP or TTMP (diluted in n‐hexane). As the *B. dorsalis* is a typical tropical insect, its courtship behavior was significantly influenced by climatic conditions. During summer, it released a greater amount of sex pheromone; conversely, in winter, its courtship activity was weaker and the release of sex pheromone was less. The time span of this study was extensive, with some assays completed in summer and others in winter. Consequently, the quantified content of sex pheromones varied significantly.

To identify the volatile compounds in the bacterial fermentation broth, a 100 µm polydimethylsiloxane (PDMS) SPME fibre (Supelco) was used to capture the volatile compounds for 30 min at room temperature by headspace sampling. Then, the compounds were analyzed by GC–MS according to the methods described above.

### Rectal Bacterial Composition

To analyze bacterial diversity in the rectum of males, rectal samples of 50 flies were collected as one sample (five replicate samples were prepared). Bacterial DNA was extracted from the rectal samples using the Bacterial Genomic DNA Extraction Kit (Tiangen, Beijing, China) according to the manufacturer's protocol. To analyze bacterial diversity in the rectum, the 16S rRNA V3‐V4 region was amplified by PCR (95 °C for 5 min, followed by 30 cycles at 95 °C for 1 min, 60 °C for 1 min, and 72 °C for 1 min, and a final extension at 72 °C for 7 min) using primers listed in Table S1 (Supporting Information). 50 µL mixture containing 10 µL of 5 × Q5@ Reaction Buffer, 10 µL of 5 × Q5@ High GC Enhancer, 1.5 µL of 2.5 mm dNTPs, 1.5 µL of each primer (10 µm), 0.2 µL of Q5@ High‐Fidelity DNA Polymerase, and 50 ng of template DNA. Related PCR reagents were from New England Biolabs, USA. Amplicons were evaluated with 2% agarose gels and purified using the AMPure XP Beads (Beckman, CA, USA) according to the manufacturer's instructions. Then, sequencing was done on a HiSeq 2500 System (Illumina). Mothur (https://www.mothur.org)^[^ ^64^ ^]^ was used to cluster tags of more than 97% identity into OTUs with the default parameters, and then the abundances of the OTUs were calculated. The taxonomic classification of OTUs was based on the annotation results of the tags according to the mode principle; i.e., the taxonomic rank that contained more than 66% of tags was considered the taxonomic rank of a specific OTU. PCA (principal component analysis) was performed in R project Vegan package (version 2.5.3) with the relative abundances of the OTUs. Considering that 16S rRNA amplicon sequencing could not reflect the absolute amount of *Bacillus* in rectum, rectal *Bacillus* absolute contents of mature male were measured by qPCR. Briefly, 5 rectums were collected as one sample. Then, the bacterial DNA in the rectum was extracted using the Bacterial Genomic DNA Extraction Kit (Tiangen, Beijing, China, http://www.tiangen.com/asset/imsupload/up0250002001571219042.pdf) according to the manufacturer's protocol. qPCR was used to estimate the differences in the absolute abundance of *Bacillus*. Primers targeting the 16S rRNA gene of *Bacillus* were designed to amplify the gene from rectal *Bacillus* (Table S1, Supporting Infirmation). Before amplification, a standard curve to quantify *Bacillus* absolute abundance was generated. Briefly, the genomic DNA of *Bacillus* ^[^ ^65^ ^]^ isolated from mature males was extracted for amplification with the primer (Table S1, Supporting Infirmation). The amplified fragment was then cloned and inserted into the pMD 18–T vector, which was then transferred into *E. coli* DH5α for propagation by heat shock at 42 °C for 90 sec. The propagated vector was then extracted with a plasmid extraction kit (DP105, TIANGEN, Beijing, China) and subjected to 10‐fold serial dilution to obtain 5 different plasmid concentrations (47.5, 4.75, 0.475, 0.0475, and 0.00475 ng µL^−1^, respecitively, measured by a Nanodrop spectrophotometer). Then, a standard curve for quantifying *Bacillus* absolute abundance was generated by amplifying the 16S rRNA of the plasmid. By referring to the standard curve, the absolute abundance of *Bacillus* in rectum was determined.

### Measurement of the Acid‐Base Environment in the Rectum

The acid‐base environment in the rectum of the flies was measured with a portable micro pH meter (APERA, SX811‐MS). Because the rectum was too small to get enough rectal suspensions for direct measurement of pH, the rectums of 10 flies were collected as one sample and placed in a 1.5 mL microcentrifuge tube containing 30 µL of sterile Milli‐Q water (pH 7). Although such method could not measure the exact rectal pH, it could distinguish acidity or alkalinity of the rectum. Then, the samples were ground in a grinding machine. The samples were then centrifuged for 10 min at 12 000 rpm. The centrifuged supernatant was transferred to another centrifuge tube. Then, the probe (LabSen241‐3SP) of the pH meter was inserted into the supernatant to determine the pH value.

### Bacillus Cultivation Experiments

Cultivation experiments were conducted in 100 mL shake flasks containing 20 mL of LB medium supplemented with membrane filtration‐sterilized threonine (0.125 g mL^−1^), glucose (0.25 g mL^−1^), and (NH_4_)_2_HPO_4_ (0.075 g mL^−1^) as the substrate to study the production of TMP and TTMP by *Bacillus* strains isolated from the male rectum.^[^ ^15^ ^]^ To test the effect of different pH values on the production of TMP and TTMP, the pH of the LB medium broth was adjusted to 6.5, 7 and 7.3 by adding sodium bicarbonate or citric acid to the medium. 200 µL *Bacillus* strains suspension (diluted by sterile water, OD: 0.98) into 20 ml LB medium and cultured aerobically at 37 °C with shaking at 200 rpm for 3 days. 3 days after, OD values of the bacteria were measured. To identify TMP and TTMP in the medium, a 100 µm polydimethylsiloxane (PDMS) SPME fibre (Supelco) was used to capture the compounds for 30 min at room temperature by headspace sampling. Then, the compounds were analyzed by GC–MS according to the methods described above. Because the identified sex pheromone was obtained by headspace extraction, the peak area was used to represent the sex pheromone abundance and the abundance of bacteria in the fermentation solution was used to normalize the abundance of sex pheromone.

### Measurement of TDH, Glucose, Threonine, Glycine, and Ammonium Contents in *Bacillus*

To measure the contents of TDH, glucose, threonine, glycine, and ammonium in *Bacillus* cultivated under different pH conditions, *Bacillus* suspensions were collected. These suspensions were first washed with PBS, then diluted to an optical density with an OD_600_ value of 2.5, and subsequently disrupted by sonication.

The measurement of TDH contents was carried out using the TDH measurement Kit (Tongwei, Shanghai, China) following the manufacturer's instructions. The quantification of TDH contents was achieved by measuring the absorbance at 450 nm with a microplate reader (Synergy H1 model from Bio‐Rad, USA).

For the measurement of glucose contents in *Bacillus*, the Glucose Assay Kit (Beyotime, China) was employed. The bacterial lysate was centrifuged at 12 000 rpm for 5 min. The glucose contents in the supernatants were quantified using a microplate reader (BioTek Synergy H1, America) according to the standard procedure.

For the quantification of threonine, glycine, and ammonium, the sample preparation for free amino acid analysis was conducted as previously described.^[^ ^40^ ^]^ The *Bacillus* lysate was centrifuged at 12 000 rpm for 15 min. The supernatants were then transferred to fresh centrifuge tubes and treated with 25 mm sulfosalicylic acid. Subsequently, the levels of threonine, glycine, and ammonium in the deproteinized supernatants were quantified using an amino acid analyzer (Hitachi L‐8900, Japan) following the instrument's standard operating protocol.

### Expression, Purification, and Catalysis of TDH

PrimerSTAR Max DNA polymerase was employed for polymerase chain reaction. The gene sequence of TDH was cloned using the genomic DNA of *Bacillus safensis* as the template. Subsequently, both the TDH fragment and pET‐32a were digested with HindIII and XhoI restriction enzymes. The digested plasmid pET‐32a and the TDH gene fragment were ligated using T4 DNA ligase, resulting in the formation of the recombinant plasmid pET‐32a‐TDH. *Escherichia coli* BL21 was then transformed with pET‐32a‐TDH for protein expression. The vector pET‐32a‐TDH isolated from *E. coli* BL21 was sequenced by Sangon Biotech (Shanghai, China). The primers and detailed PCR conditions utilized in this study were presented in Table S1 (Supporting Infirmation).

The recombinant *E. coli* BL21/pET‐32a‐TDH strain was cultured in LB medium supplemented with 100 µg mL^−1^ ampicillin at 37 °C with shaking at 200 revolutions per minute (rpm). Once the optical density at 600 nm (OD_600_) reached 0.6, 0.5 mm isopropyl‐β‐D‐thiogalactoside (IPTG) was added to induce protein expression. The induction was carried out at 17 °C with shaking at 200 rpm for 2 h. The cells were harvested by centrifugation at 10 000 rpm for 20 min, washed twice with 0.85% NaCl solution (centrifuged at 10 000 rpm for 5 min), resuspended in buffer A (20 mm Na_3_PO_4_•12H_2_O, 500 mm NaCl, pH 8.0), and subsequently disrupted by sonication in an ice‐water bath. The supernatant was then collected by centrifugation at 4 °C at 10 000 rpm for 30 min. The purification of TDH was accomplished by elution with a gradient of buffer B (20 mm Na_3_PO_4_•12H_2_O, 500 mm NaCl, 400 mm imidazole, pH 8.0) using an ÄKTA purifier equipped with a HisTrap HP affinity column (5 mL) (GE Healthcare, Piscataway, NJ, USA). Purified TDH was detected though sodium dodecyl sulfate‐polyacrylamide gel electrophoresis (SDS‐PAGE).

Catalysis assays were conducted in 50 mL shake flasks, each containing 10 mL of buffer B supplemented with L‐threonine (20 mm), NAD⁺ (2 mm), acetoin (20 mm), and (NH_4_)_2_HPO_4_ (20 mm) as substrates. The aim was to investigate the production of TMP and TTMP by the purified TDH from *E. coli* BL21/pET‐32a‐TDH. To assess the impact of pH on the production of TMP and TTMP, the buffer B medium was adjusted to pH values of 6.5, 7.0, and 7.3 using HCl. The catalysis reactions were incubated aerobically at 37 °C with shaking at 200 revolutions per minute (rpm) for 24 h. To identify TMP and TTMP in the medium, a 100 µm polydimethylsiloxane (PDMS) solid‐phase microextraction (SPME) fibre (Supelco) was employed. The compounds were captured for 30 min at room temperature via headspace sampling. Subsequently, the compounds were analyzed by gas chromatography‐mass spectrometry (GC–MS) following the methods described previously. Since the identified sex pheromone was obtained through headspace extraction, the peak area was utilized to represent the abundance of the sex pheromone.

### Comparative Transcriptomic Analysis and Gene Identification

To identify the genes that regulate rectal pH, the rectal transcriptomes of males at different developmental stages (0, 3, 6, 9 and 12‐day‐old males) and at different times of day (0:00, 4:00, 8:00, 12:00, 16:00, and 20:00) were sequenced. For each sample, 50 rectums were dissected for RNA extraction using the TRIzol Reagent (Invitrogen) according to the manufacturer's instructions. In addition, five replicates were included for each time point. Then, paired‐end RNA‐seq libraries were prepared by following Illumina's mRNA library construction protocol. Briefly, the mRNA was enriched using mRNA Capture Beads. After purification with beads, the mRNA was fragmented using high temperatures. The fragmented mRNA was then used as a template to synthesize the first strand of cDNA in a reverse transcription enzyme mixture system. While synthesizing the second strand of cDNA, end repair, and A‐tailing were completed. Next, adapters were ligated, and Hieff NGS DNA selection beads were used for purification to select target fragments. Then, the libraries were sequenced on an Illumina HiSeq2000 platform (Illumina, USA). All sequencing of samples at different development stages or at different times of a day was accomplished in a single run. Then, FASTQ files of the raw reads were produced and sorted by barcodes for further analysis. Prior to assembly, paired‐end raw reads from each cDNA library were processed to remove adaptors, low‐quality sequences (Q < 20), and reads contaminated with microbes by fastp (version 0.18.0).^[^ ^66^ ^]^ An index of the reference genome (GCF_02 337 3825.1 in NCBI) of *B. dorsalis* was built, and paired‐end clean reads were mapped to the reference genome using HISAT2. 2.4 with “‐rna‐strandness RF” and other parameters set as a default.^[^ ^67^ ^]^ The mapped reads of each sample were assembled by using StringTie v1.3.1^[^ ^68^ ^]^ in a reference based approach. To evaluate transcript expression abundances, StringTie v1.3.1 was further used to calculate the normalized gene expression value (FPKM) with parameters set as a default.^[^ ^69^ ^]^ Correlation analysis was performed by “corrplot” package in R with the gene expression profile of each sample. Correlations of two samples were used to evaluate the similarity of overall gene expression. The closer the correlation coefficient was to 1, the greater the similarity between two samples. Then, differential gene expression analysis was performed with the DESeq2 R command package.^[^ ^70^ ^]^ Parameters were set as genes/transcripts with a false discovery rate (FDR) less than 0.01 and an absolute fold change ≥ 2 were considered differentially expressed genes/transcripts (DE genes).

### Validation of the Expression of the Identified Genes

qRT‐PCR analysis was used to validate the expression of CA genes in the head, thorax, abdomen, gut, and rectum of mature males. Total RNA was extracted from these tissues using the TRIzol Reagent (Invitrogen) according to the manufacturer's instructions. Then, cDNA was synthesized with a One‐Step gDNA Removal and cDNA Synthesis SuperMix Kit (TransGen Biotech, Beijing, China) using the extracted RNA. Then, a PerfectStarTM Green qPCR SuperMix Kit (TransGen Biotech, Beijing, China) was used to perform quantitative real‐time PCR to compare the gene expression levels. Gene‐specific primers (Table S1, Supporting Infirmation) were designed with primer BLAST in the NCBI database. The *α‐tubulin* and *actin* genes were used as reference genes.^[^ ^71^ ^]^ The PCR procedure was performed according to the manufacturers’ instructions. Five biological replicates were performed. The expression of *CAh* genes in the rectum of mature males at different developmental stages and at different times of day was determined by the same method described above.

### Phylogenetic Sequence Analysis

Phylogenetic analysis using amino acid sequence alignments for CAh sequences identified from the fruitfly (*Drosophila melanogaster*), mosquito (*Aedes aegypti*), housefly (*Musca domestica*), bee (*Apis mellifera*), beetle (*Tribolium castaneum*), and moth (*Manduca sexta*) genomes. CAh amino acid sequence analysis was performed with MEGA11,^[^ ^72^ ^]^ and maximum likelihood (ML) tree reconstruction was performed using the Poisson model and uniform rates. The ML heuristic search was performed with the nearest neighbor‐change method, and the initial tree was selected by applying the neighbor joining method to a matrix of pairwise distances estimated using the JTT method. The accuracy of the tree was tested with bootstrapping using 500 replicates.

### *CAh* Enzyme Activity Measurement

Because *CAh* were involved in balancing the pH by catalyzing the reversible hydration of carbon dioxide,^[^ ^47^ ^]^ changes in pH in the rectum may be correlated with the activity of CAh enzyme. Therefore, the activity of *CAh* enzyme in the rectum was measured. To measure *CAh* activity in the rectum of males, 10 rectums of mature males were dissected and collected. Then, *CAh* activities were measured using the CAh Activity Kit (Tongwei, Shanghai, China) according to the manufacturer's protocol. The *CAh* activity in the samples was quantified with a microplate reader (BioTek Synergy H1, America) according to the standard method.

### RNA Interference

Double‐stranded RNA (dsRNA) primers (Table S1, Supporting Information) tailed with the T7 promoter sequence were designed using the CDSs of CAs as templates (the accession numbers in NCBI of CAh1, CAh7, βCAh7, CAh2‐1 and CAh2 are XM_04 944 8952, XM_01 120 3355, XM_04 946 1178, XM_01 121 3342, and XM_04 944 8380, respectively) (Figure S11, Supporting Information). To avoid off‐target effect in RNAi, the online tool (Dsomg.sysu.edu.cn) was used to perform off‐target prediction of RNAi target sites in *B. dorsalis* by setting the threshold of off‐target bases to 19 bp.^[^ ^73^ ^]^ Then, a MEGAscript RNAi Kit (Thermo Fisher Scientific, United States) was used to synthesize and purify dsRNA according to the manufacturer's instructions. The GFP gene (GenBank accession number: AHE38523) was used as the RNAi negative control. To knock down the target gene in males, 0.5 µL (500 ng µL^−1^) of dsRNA was injected into the abdomen of 12‐day‐old males. Flies injected with dsGFP were used as a negative control. After 24 h, the knockdown efficiency of the genes was checked via qRT–PCR following the method used for validating the expression of the genes described above. Then, the *CAh* enzyme activity, rectal pH, sex pheromone level and mating ability of the males were compared.

### Data Analysis

The statistical methods used in the study were indicated in the figure legends. Differences were considered significant when *p* < 0.05. All the data were analyzed using GraphPad Prism version 10 (GraphPad Software, La Jolla, California, USA; www.graphpad.com). Prior to heat mapping, the data in each column or row was standardized by means of the Z‐score standardization method.^[^ ^74^ ^]^

## Conflict of Interest

The authors declare no conflict of interest.

## Supporting information



Supporting Information

## Data Availability

All the data needed to evaluate the conclusions in the paper are presented in the paper and/or the Supplementary Materials. RNA‐sequencing and 16S rRNA amplicon sequencing data have been deposited in the Genome Sequence Read Archive Database of the National Genomics Data Center (PRJCA010560, PRJCA015618, PRJCA024486, and PRJCA024527).

## References

[1] X. Chen , R. D'Souza , S.‐T. Hong , Protein Cell 2013, 4, 403. 23686721 10.1007/s13238-013-3017-x PMC4875553

[2] P. D. Cani , M. Van Hul , C. Lefort , C. Depommier , M. Rastelli , A. Everard , Nat. Metab. 2019, 1, 34. 32694818 10.1038/s42255-018-0017-4

[3] D. Li , P. Wang , P. Wang , X. Hu , F. Chen , Biotechnol. Adv. 2016, 34, 1210. 27592384 10.1016/j.biotechadv.2016.08.003

[4] E. M. Janson , J. O. Stireman , M. S. Singer , P. Abbot , Evolution 2008, 62, 997. 18298649 10.1111/j.1558-5646.2008.00348.x

[5] S. C. Shin , S.‐H. Kim , H. You , B. Kim , A. C. Kim , K.‐A. Lee , J.‐H. Yoon , J. I.‐H. Ryu , W.‐J. Lee , Science 2011, 334, 670. 22053049 10.1126/science.1212782

[6] R. I. Mackie , Integr. Comp. Biol. 2002, 42, 319. 21708724 10.1093/icb/42.2.319

[7] K. D. Kohl , R. B. Weiss , J. Cox , C. Dale , M. D. Dearing , Ecol. Lett. 2014, 17, 1238. 25040855 10.1111/ele.12329

[8] T. Z. Jing , F. H. Qi , Z. Y. Wang , Microbiome 2020, 8, 38. 32178739 10.1186/s40168-020-00823-y PMC7077154

[9] T. Rigaud , P. S. Pennings , P. Juchault , J. Invertebr. Pathol. 2001, 77, 251. 11437528 10.1006/jipa.2001.5026

[10] M. McFall‐Ngai , M. G. Hadfield , T. C. G. Bosch , H. V. Carey , T. Domazet‐Loso , A. E. Douglas , N. Dubilier , G. Eberl , T. Fukami , S. F. Gilbert , U. Hentschel , N. King , S. Kjelleberg , A. H. Knoll , N. Kremer , S. K. Mazmanian , J. L. Metcalf , K. Nealson , N. E. Pierce , J. F. Rawls , A. Reid , E. G. Ruby , M. Rumpho , J. G. Sanders , D. Tautz , J. J. Wernegreen , Proc. Natl. Acad. Sci. U. S. A. 2013, 110, 3229. 23391737 10.1073/pnas.1218525110 PMC3587249

[11] R. D. Heijtz , S. Wang , F. Anuar , Y. Qian , B. Björkholm , A. Samuelsson , M. L. Hibberd , H. Forssberg , S. Pettersson , Proc. Natl. Acad. Sci. U. S. A. 2011, 108, 3047. 21282636 10.1073/pnas.1010529108 PMC3041077

[12] J. F. Cryan , T. G. Dinan , Nat. Rev. Neurosci. 2012, 13, 701. 22968153 10.1038/nrn3346

[13] J. M. Brand , J. W. Bracke , A. J. Markovetz , D. L. Wood , L. E. Browne , Nature 1975, 254, 136. 804144 10.1038/254136a0

[14] R. J. Dillon , C. T. Vennard , A. K. Charnley , Nature 2000, 403, 851. 10.1038/35002669 10706273

[15] L. Ren , Y. G. Ma , M. X. Xie , Y. Y. Lu , D. F. Cheng , Curr. Biol. 2021, 31, 2220. 33740424 10.1016/j.cub.2021.02.046

[16] M.‐Y. Zhang , X.‐H. Zhang , X.‐Y. Wang , Y. U.‐L. Liu , J.‐H. An , D.‐H. Wang , Z.‐G. Cai , R. Hou , Front. Microbiol. 2023, 14, 1234676. 37692393 10.3389/fmicb.2023.1234676 PMC10485365

[17] A. Lize , R. McKay , Z. Lewis , Trends Ecol. Evol. 2013, 28, 325 . 23141109 10.1016/j.tree.2012.10.013

[18] A. Wada‐Katsumata , L. Zurek , G. Nalyanya , W. L. Roelofs , A. Zhang , C. Schal , Proc. Natl. Acad. Sci. U. S. A. 2015, 112, 15678. 26644557 10.1073/pnas.1504031112 PMC4697420

[19] H. Qiao , I. W. Keesey , B. S. Hansson , M. Knaden , J. Exp. Biol. 2019, 222, jeb192500. 30679242 10.1242/jeb.192500

[20] M. Groussin , F. Mazel , E. J. Alm , Cell Host Microbe. 2020, 28, 12 . 32645351 10.1016/j.chom.2020.06.013

[21] H. Itoh , S. Jang , K. Takeshita , T. Ohbayashi , N. Ohnishi , X.‐Y. Meng , Y. Mitani , Y. Kikuchi , Proc. Natl. Acad. Sci. U. S. A. 2019, 116, 22673. 31636183 10.1073/pnas.1912397116 PMC6842582

[22] S. Jang , Y. Matsuura , K. Ishigami , P. Mergaert , Y. Kikuchi , Front. Physiol. 2023, 13, 1071987. 36685208 10.3389/fphys.2022.1071987 PMC9846216

[23] J. K. Kim , N. H. Kim , H. A. Jang , Y. Kikuchi , C.‐H. Kim , T. Fukatsu , B. L. Lee , Appl. Environ. Microbiol. 2013, 79, 7229. 24038695 10.1128/AEM.02152-13 PMC3837730

[24] T. Ohbayashi , P. Mergaert , Y. Kikuchi , Mechanisms Underlying Microbial Symbiosis, *Advances in Insect Physiology* , Springer Nature, Cham, Switzerland 2020, Vol. 58.

[25] R. M. Moll , W. S. Romoser , M. C. Modrzakowski , A. C. Moncayo , K. Lerdthusnee , J. Med. Entomol. 2001, 38, 29. 11268687 10.1603/0022-2585-38.1.29

[26] T. Köhler , C. Dietrich , R. H. Scheffrahn , A. Brune , Appl. Environ. Microbiol. 2012, 78, 4691. 22544239 10.1128/AEM.00683-12 PMC3370480

[27] C. Dietrich , T. Köhler , A. Brune , Appl. Environ. Microbiol. 2014, 80, 2261. 24487532 10.1128/AEM.04206-13 PMC3993134

[28] C. P. Hopper , L. K. De La Cruz , K. V. Lyles , L. K. Wareham , J. A. Gilbert , Z. Eichenbaum , M. Magierowski , R. K. Poole , J. Wollborn , B. Wang , Chem. Rev. 2020, 120, 13273. 33089988 10.1021/acs.chemrev.0c00586

[29] L. Kesnerová , O. Emery , M. Troilo , J. Liberti , B. Erkosar , P. Engel , ISME J. 2020, 14, 801. 31836840 10.1038/s41396-019-0568-8 PMC7031341

[30] A. Quinn , Y. El Chazli , S. Escrig , J. Daraspe , N. Neuschwander , A. McNally , C. Genoud , A. Meibom , P. Engel , Nat. Microbiol. 2024, 9, 477. 38225461 10.1038/s41564-023-01572-y PMC11343714

[31] P. Engel , N. A. Moran , FEMS Microbiol. Rev. 2013, 37, 699. 23692388 10.1111/1574-6976.12025

[32] H. M. Appel , M. M. Martin , J. Chem. Ecol. 1990, 16, 3277 . 24263429 10.1007/BF00982098

[33] J. F. Harrison , Annu. Rev. Entomol. 2001, 46, 221. 11112169 10.1146/annurev.ento.46.1.221

[34] A. Brune , M. Friedrich , Curr. Opin. Microbiol. 2000, 3, 263. 10851155 10.1016/s1369-5274(00)00087-4

[35] H. LIU , D.‐J. ZHANG , Y.‐J. XU , L. WANG , D.‐F. CHENG , Y.‐X. QI , L. ZENG , Y. LU , J. Integr. Agric. 2019, 18, 771.

[36] S. Jaffar , S. A. H. Rizvi , Y. Lu , Horticulturae 2023, 9, 1004.

[37] J. L. Hoskins , P. Rempoulakis , M. M. Stevens , B. C. Dominiak , Insects 2023, 14, 801 . 37887813 10.3390/insects14100801 PMC10607784

[38] X. Zhao , X. Zhang , Z. Chen , Z. Wang , Y. Lu , D. Cheng , Front. Microbiol. 2018, 9, 114. 29449838 10.3389/fmicb.2018.00114 PMC5799270

[39] S. Gui , B. Yuval , T. Engl , Y. Lu , D. Cheng , Elife 2023, 12, 83469. 10.7554/eLife.83469 PMC9908074 36656757

[40] J. Chen , Y. Jiang , Z. Gao , J. Dai , C. Jia , Y. Lu , D. Cheng , Adv. Sci. 2024, 11, 2407353 . 10.1002/advs.202407353 PMC11600207 39377305

[41] Z. Gao , M. Xie , S. Gui , M. He , Y. Lu , L. Wang , J. Chen , G. Smagghe , J. Gershenzon , D. Cheng , ISME J. 2023, 17, 1741. 37550382 10.1038/s41396-023-01488-9 PMC10504272

[42] X. Li , Z. Wang , J. Chen , H. Teng , X. Yang , L. Ye , Y. Jiang , H. Chen , D. Cheng , Y. Lu , Cell Rep. 2024, 43, 115030. 39616614 10.1016/j.celrep.2024.115030

[43] C. G. Corrales‐Maldonado , I. Vargas‐Arispuro , R. Pérez‐Morales , M. Á. Martínez‐Téllez , E. Aispuro‐Hernández , G. D. Ávila‐Quezada , Appl. Entomol. Zool. 2024, 59, 237.

[44] M. Saranya , J. S. Kennedy , R. Anandham , A. Manikandan , Appl. Entomol. Zool. 2022, 57, 323.

[45] G. Li , X. Zheng , Y. Zhu , Y. Long , X. Xia , Environ. Microbiol. 2022, 24, 4049. 35191580 10.1111/1462-2920.15934

[46] L. J. Zhang , Y. L. Cao , J. N. Tong , Y. Xu , Appl. Environ. Microbiol. 2019, 85, 01807. 10.1128/AEM.01807-19 PMC6881806 31585995

[47] A. Aspatwar , M. E. E. Tolvanen , H. Barker , L. Syrjänen , S. Valanne , S. Purmonen , A. Waheed , W. S. Sly , S. Parkkila , Physiol. Rev. 2022, 102, 1327. 35166161 10.1152/physrev.00018.2021

[48] S. Shanbhag , S. Tripathi , J. Exp. Biol. 2009, 212, 1731. 19448082 10.1242/jeb.029306

[49] G. Overend , Y. Luo , L. Henderson , A. E. Douglas , S. A. Davies , J. A. T. Dow , Sci. Rep. 2016, 6, 27242. 27250760 10.1038/srep27242 PMC4890030

[50] R. Z. Emameh , L. Syrjaenen , H. Barker , C. T. Supuran , S. Parkkila , J. Enzyme Inhib. Med. Chem. 2015, 30, 505. 25198895 10.3109/14756366.2014.944178

[51] M. del Pilar Corena , T. J. Seron , H. K. Lehman , J. D. Ochrietor , A. Kohn , C. Tu , P. J. Linser , J. Exp. Biol. 2002, 205, 591 . 11907049 10.1242/jeb.205.5.591

[52] L. B. Schultze , A. Maldonado , A. Lussi , A. Sculean , S. Eick , Monogr. Oral Sci. 2021, 29, 19 . 33427214 10.1159/000510196

[53] M. Rektorschek , D. Weeks , G. Sachs , K. Melchers , Gastroenterology 1998, 115, 628. 9721160 10.1016/s0016-5085(98)70142-8

[54] T. Millat , H. Janssen , G. J. Thorn , J. R. King , H. Bahl , R.‐J. Fischer , O. Wolkenhauer , Appl. Microbiol. Biotechnol. 2013, 97, 6451. 23640360 10.1007/s00253-013-4860-7

[55] R. J. Palframan , G. R. Gibson , R. A. Rastall , Anaerobe 2002, 8, 287. 16887671 10.1006/anae.2002.0434

[56] G. Sharon , D. Segal , J. M. Ringo , A. Hefetz , I. Zilber‐Rosenberg , E. Rosenberg , Proc. Natl. Acad. Sci. U. S. A. 2010, 107, 20051 . 21041648 10.1073/pnas.1009906107 PMC2993361

[57] Q. J. Cao , Y. Zhao , T. M. Koski , H. P. Li , J. H. Sun , Insect Sci. 2024, 31, 225. 37221982 10.1111/1744-7917.13210

[58] S. Caccia , M. Casartelli , G. Tettamanti , Cell Tissue Res. 2019, 377, 505. 31359140 10.1007/s00441-019-03076-w

[59] M. Holtof , C. Lenaerts , D. Cullen , J. Vanden Broeck , Cell Tissue Res. 2019, 377, 397. 31037358 10.1007/s00441-019-03031-9

[60] M. T. Naseem , R. Beaven , T. Koyama , S. Naz , S.‐Y. Su , D. P. Leader , D. A. Klaerke , K. Calloe , B. Denholm , K. V. Halberg , Proc. Natl. Acad. Sci. U. S. A. 2023, 120, 2217084120. 10.1073/pnas.2217084120 PMC10068851 36943876

[61] H. Koenig , L. Li , J. Froehlich , Appl. Microbiol. Biotechnol. 2013, 97, 7943. 23900801 10.1007/s00253-013-5119-z

[62] M. E. Scharf , Curr. Opin. Insect Sci. 2020, 41, 79. 32823202 10.1016/j.cois.2020.07.007

[63] A.‐G. Planson , V. Sauveplane , E. Dervyn , M. Jules , Biochimi. Biophys. Acta 2020, 1863, 194502. 10.1016/j.bbagrm.2020.194502 32044462

[64] S. D. Hiltemann , S. A. Boers , P. J. van der Spek , R. Jansen , J. P. Hays , A. P. Stubbs , Gigascience 2019, 8, giy166 . 30597007 10.1093/gigascience/giy166 PMC6377400

[65] L. Ren , Y. Ma , M. Xie , Y. Lu , D. Cheng , Curr. Biol. 2021, 31, 2220. 33740424 10.1016/j.cub.2021.02.046

[66] S. Chen , Y. Zhou , Y. Chen , J. Gu , Bioinformatics 2018, 34, i884. 30423086 10.1093/bioinformatics/bty560 PMC6129281

[67] D. Kim , B. Langmead , S. L. Salzberg , Nat. Methods 2015, 12, 357. 25751142 10.1038/nmeth.3317 PMC4655817

[68] M. Pertea , G. M. Pertea , C. M. Antonescu , T.‐C. Chang , J. T. Mendell , S. L. Salzberg , Nat. Biotechnol. 2015, 33, 290. 25690850 10.1038/nbt.3122 PMC4643835

[69] M. Pertea , D. Kim , G. M. Pertea , J. T. Leek , S. L. Salzberg , Nat. Protoc. 2016, 11, 1650. 27560171 10.1038/nprot.2016.095 PMC5032908

[70] H. Varet , L. Brillet‐Gueguen , J.‐Y. Coppee , M.‐A. S. Dillies , PLoS One 2016, 0157022, 11. 10.1371/journal.pone.0157022 PMC4900645 27280887

[71] G.‐M. Shen , H.‐B. Jiang , X.‐N. Wang , J.‐J. Wang , BMC Mol. Biol. 2010, 11, 76. 20923571 10.1186/1471-2199-11-76 PMC2972281

[72] K. Tamura , G. Stecher , S. Kumar , Mol. Biol. Evol. 2021, 38, 3022. 33892491 10.1093/molbev/msab120 PMC8233496

[73] M. M. Kulkarni , M. Booker , S. J. Silver , A. Friedman , P. Hong , N. Perrimon , B. Mathey‐Prevot , Nat. Methods 2006, 3, 833. 16964256 10.1038/nmeth935

[74] L. A. Shalabi , Z. Shaaban , B. Kasasbeh , J. Comput. Sci. 2006, 2, 735.

