# Supplementary material for: Alkaline–Acid Intestine Environment Controlled by A Carbonic Anhydrase Gene Influences Synthesis of Sex Pheromone by Symbionts
Source: Adv Sci (Weinh). 2025 Sep 14;12(43):e11723. doi: 10.1002/advs.202511723 (PMC12631853; doi:10.1002/advs.202511723)
Supplement: Supplementary file 1 — Supporting Information [file ADVS-12-e11723-s001.docx]

**
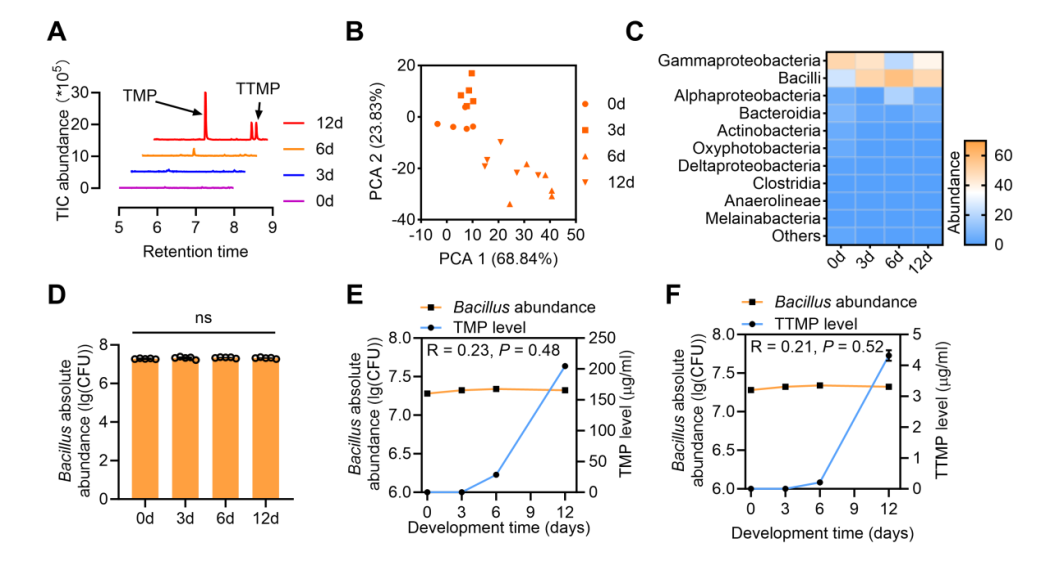
**

**Fig. S1. Change of sex pheromone and bacteria in rectum of male at different developmental stages.**

(**A**) GC-MS ion chromatograms of male rectum extracts at 20:00 across different developmental stages. To prevent the peaks of sex pheromones from different treatments from overlapping one another, the chromatograms were shifted.

(**B**) Comparison of rectal bacterial diversity between male at 20:00 across different developmental stages.

(**C**) Rectal bacteria abundance in male at 20:00 across different developmental stages.

(**D**) Absolute content of rectal *Bacillus* in male at 20:00 across different developmental stages. (n = 5, *F*_(3,16)_ = 1.787, *P* = 0.1901, one-way ANOVA).

(**E**) Correlation between the levels of TMP and the abundance of rectal *Bacillus* in males at 20:00 across different developmental stages.

(**F**) Correlation between the levels of TTMP and the abundance of rectal *Bacillus* in males at 20:00 across different developmental stages.

**
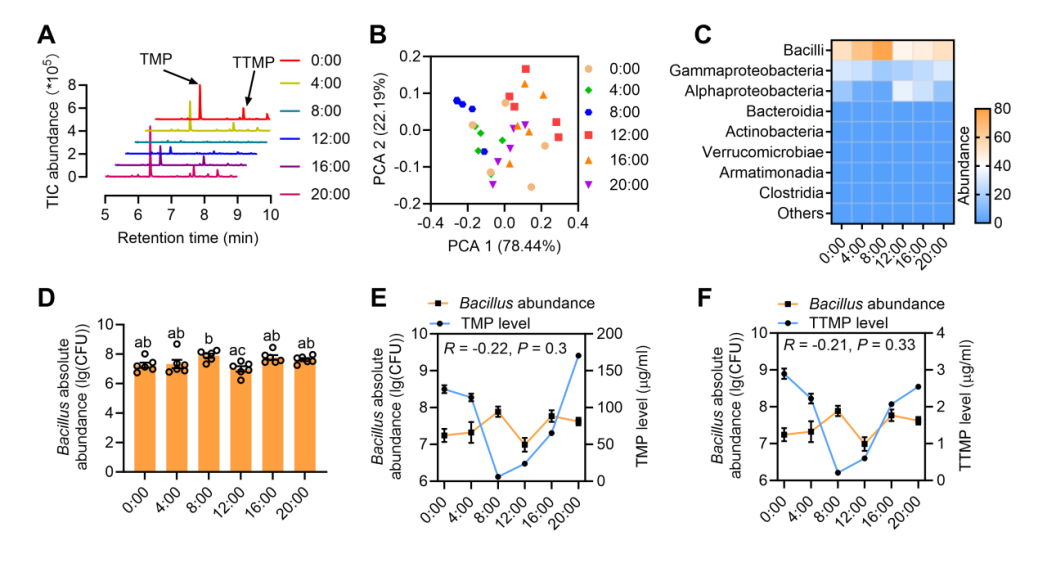
**

**Fig. S2. Change of sex pheromone and bacteria in rectum of mature male at different time of day.**

(**A**) GC-MS ion chromatograms of male rectum extracts at different time of a day.

(**B**) Male rectal bacterial diversity comparison at different time of a day. To prevent the peaks of sex pheromones from different treatments from overlapping one another, the chromatograms were shifted.

(**C**) Rectal bacteria abundance in male at different time of day.

(**D**) Rectal *Bacillus* absolute abundance comparison in male at different time of a day (n = 6, *F*_(5,30)_ = 3.455, *P* = 0.0139, one-way ANOVA). Different letters above the error bars indicate significant differences at the 0.05 level analyzed by ANOVA followed by Tukey’s test.

(**E**) Correlation between the levels of TMP and the abundance of rectal *Bacillus* in males at different times of day.

(**F**) Correlation between the levels of TTMP and the abundance of rectal *Bacillus* in males at different times of day.

**
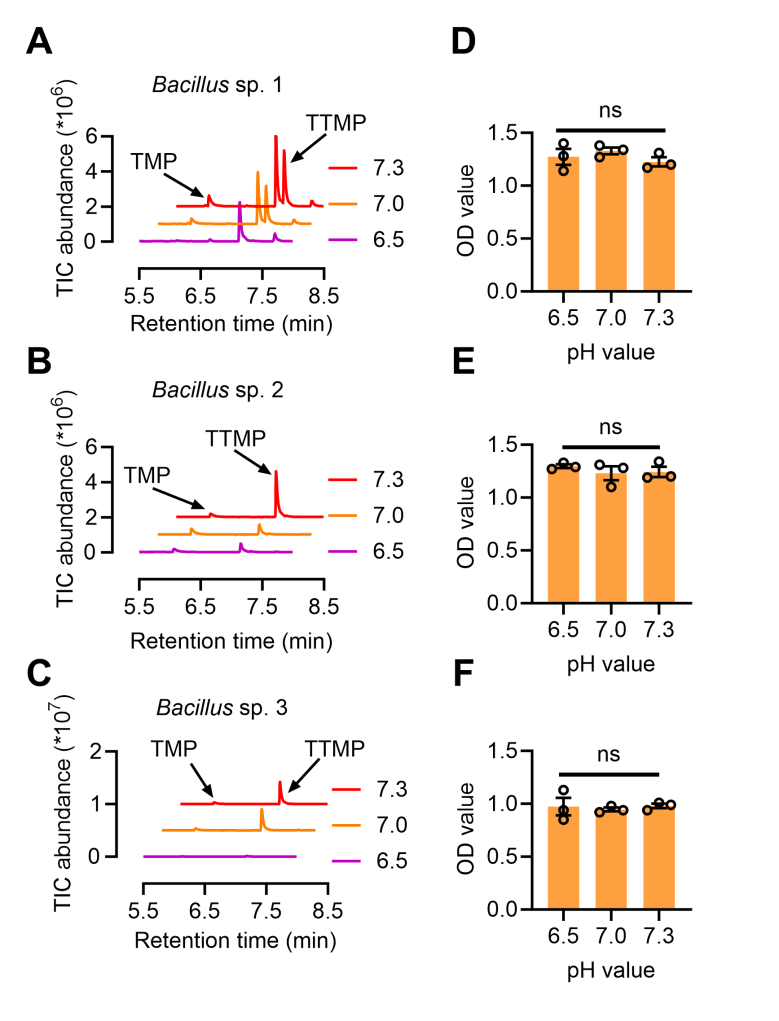
**

**Fig. S3. Influence of PH on sex pheromone synthesis activity of rectal bacteria.**

(**A**) GC-MS ion chromatograms of volatiles produced by *Bacillus* sp. 1 in different pH cultures.

(**B**) GC-MS ion chromatograms of volatiles produced by *Bacillus* sp. 2 in different pH cultures.

(**C**) GC-MS ion chromatograms of volatiles produced by *Bacillus* sp. 3 in different pH cultures.

(**D**) OD value of *Bacillus* sp. 1 3 days after culturing in different pH medium (n = 3, *F*_(2,6)_ = 0.9463, *P* = 0.4393, one-way ANOVA).

(**E**) OD value of *Bacillus* sp. 2 3 days after culturing in different pH medium (n = 3, *F*_(2,6)_ = 0.1235, *P* = 0.886, one-way ANOVA).

(**F**) OD value of *Bacillus* sp. 2 3 days after culturing in different pH medium (n = 3, *F*_(2,6)_ = 5367, *P* = 0.6103, one-way ANOVA).

In (**A**), (**B**) and (**C**), to prevent the peaks of sex pheromones from different treatments from overlapping one another, the chromatograms were shifted.


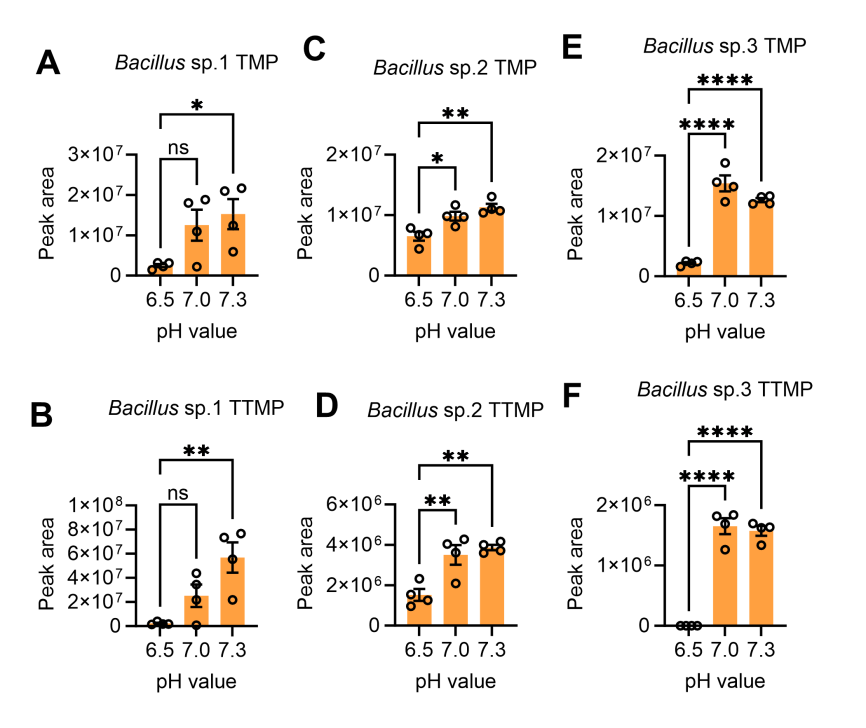


**Fig. S4. Abundance of sex pheromone produced by rectal *Bacillus* in different PH cultures.**

(**A**) Abundance of TMP produced by *Bacillus* sp. 1 in different PH cultures (n = 4, *F*_(2,9)_ = 4.654, *P* = 0.0409, one-way ANOVA).

(**B**) Abundance of TTMP produced by *Bacillus* sp. 1 in different PH cultures (n = 4, *F*_(2,9)_ = 9.205, *P* =0.0067, one-way ANOVA).

(**C**) Abundance of TMP produced by *Bacillus* sp. 2 in different PH cultures (n = 4, *F*_(2,9)_ = 12.56, *P* = 0.0025, one-way ANOVA).

(**D**) Abundance of TTMP produced by *Bacillus* sp. 2 in different PH cultures (n = 4, *F*_(2,9)_ = 13.98, *P* = 0.0017, one-way ANOVA).

(**E**) Abundance of TMP produced by *Bacillus* sp. 3 in different PH cultures (n = 4, *F*_(2,9)_ = 75.77, *P* < 0.0001, one-way ANOVA).

(**F**) Abundance of TTMP produced by *Bacillus* sp. 3 in different PH cultures (n = 4, *F*_(2,9)_ = 107, *P* < 0.0001, one-way ANOVA).


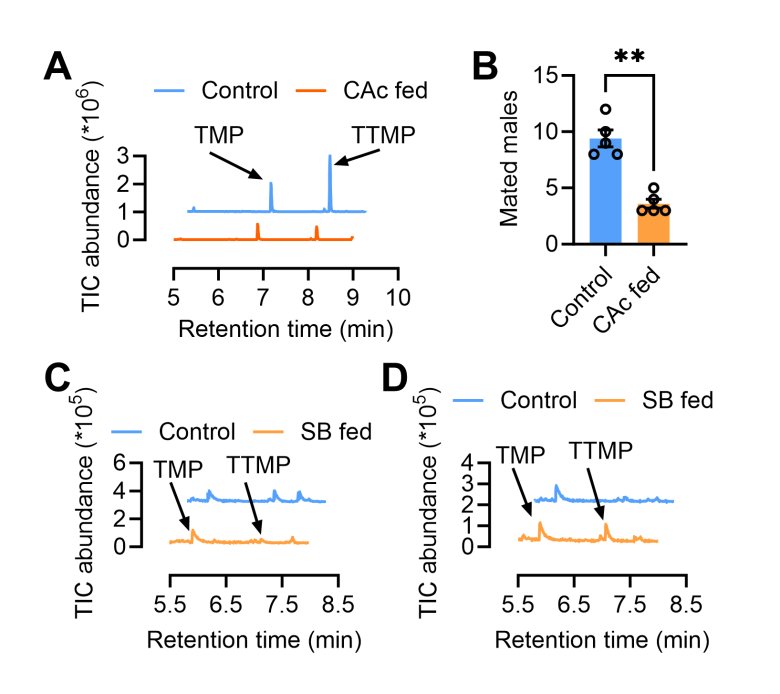


**Fig. S5. Influence of citric acid (CAc) and sodium bicarbonate (SB) feeding on sex pheromone synthesis.**

(**A**) GC-MS ion chromatograms of CAc fed mature male rectum extracts. To prevent the peaks of sex pheromones from different treatments from overlapping one another, the chromatograms were shifted.

(**B**) Influence of CAc feeding on male mating ability (n = 5, *P* = 0.0019, paired sample Student’s *t* test).

(**C**) GC-MS ion chromatograms of SB fed 6-day-old male rectum extracts. To prevent the peaks of sex pheromones from different treatments from overlapping one another, the chromatograms were shifted.

(**D**) GC-MS ion chromatograms of SB fed mature male rectum extracts.

In the figures, pH and sex pheromone level were measured at 20:00.

**
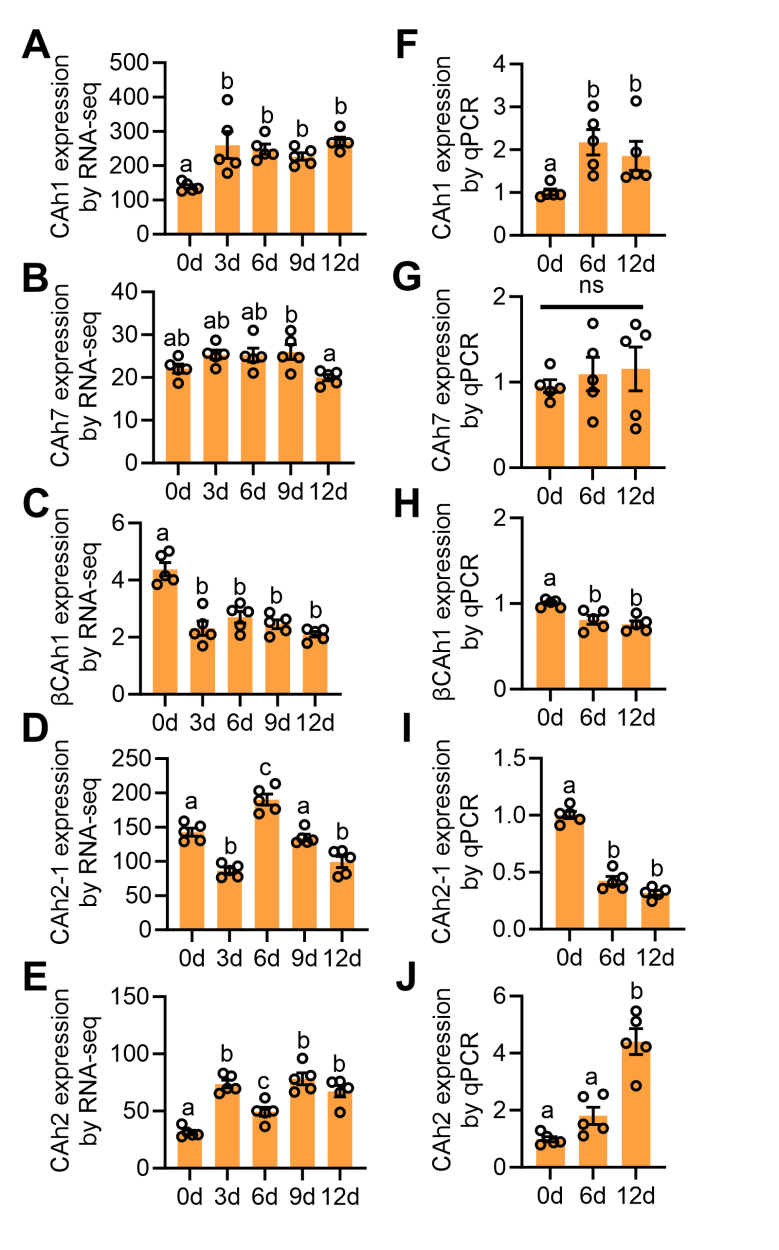
**

**Fig. S6. Expression of CAh genes in male rectum at different developmental stages.**

(**A**) Expression of CAh1 in rectum of males at different development stages determined by RNA-seq (n = 5, *F*_(4,20)_ = 6.925, *P* = 0.0011).

(**B**) Expression of CAh7 in rectum of males at different development stages determined by RNA-seq (n = 5, *F*_(4,20)_ = 3.951, *P* = 0.016).

(**C**) Expression of βCAh1 in rectum of males at different development stages determined by RNA-seq (n = 5, *F*_(4,20)_ = 21.99, *P* < 0.0001).

(**D**) Expression of CAh2-1 in rectum of males at different development stages determined by RNA-seq (n = 5, *F*_(4,20)_ = 40.45, *P* < 0.0001).

(**E**) Expression of CAh2 in rectum of males at different development stages determined by RNA-seq (n = 5, *F*_(4,20)_ = 22.87, *P* < 0.0001).

(**F**) Expression of CAh1 in rectum of males at different development stages determined by qPCR (n = 5, *F*_(2,12)_ = 5.243, *P* = 0.0231).

(**G**) Expression of CAh7 in rectum of males at different development stages determined by qPCR (n = 5, *F*_(2,12)_ = 0.2909, *P* = 0.7527).

(**H**) Expression of βCAh1 in rectum of males at different development stages determined by qPCR (n = 5, *F*_(2,12)_ = 10.91, *P* = 0.002).

(**I**) Expression of CAh2-1 in rectum of males at different development stages determined by qPCR (n = 5, *F*_(2,12)_ = 140.8, *P* < 0.0001).

(**J**) Expression of CAh2 in rectum of males at different development stages determined by qPCR (n = 5, *F*_(2,12)_ = 31.65, *P* < 0.0001).

Different letters above the error bars indicate significant differences at the 0.05 level analyzed by ANOVA followed by Tukey’s test. In the figures, gene expression levels were measured at 20:00.


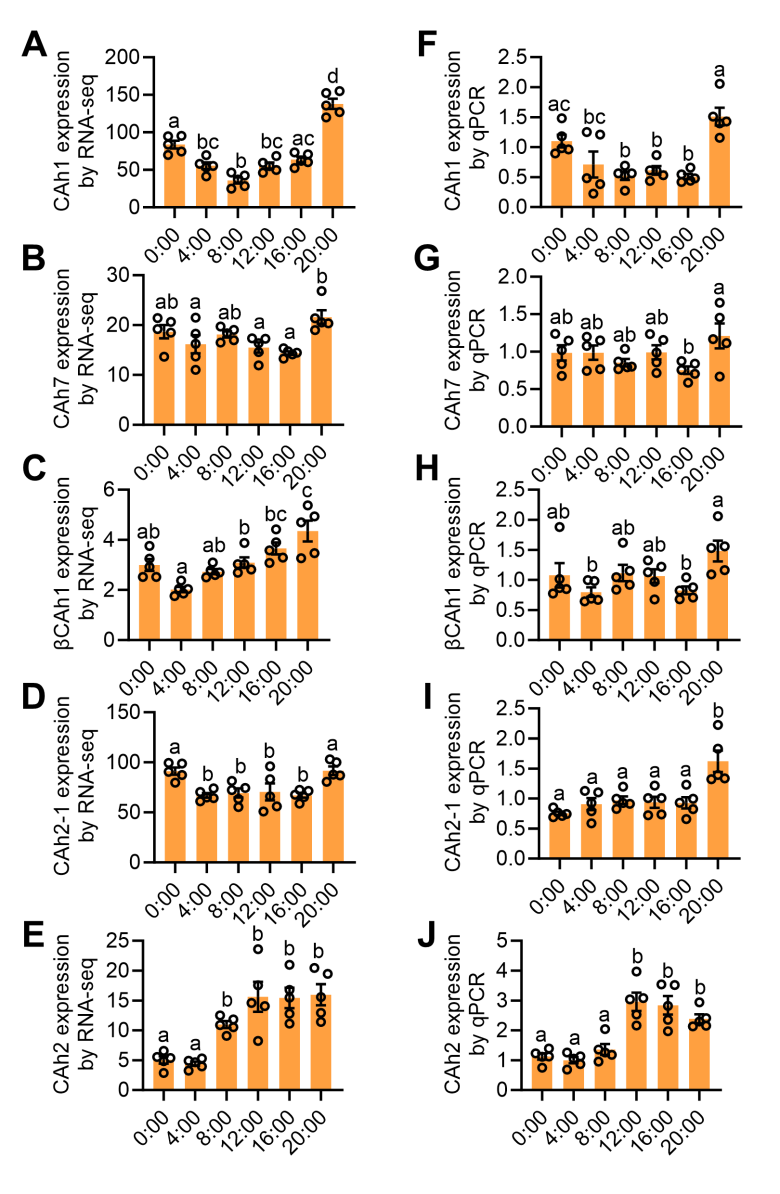


**Fig. S7. Expression of CAh genes in male rectum at different time of day.**

(**A**) Expression of CAh1 in rectum of males at different time of day determined by RNA-seq (n = 5, *F*_(5,24)_ = 54.54, *P* < 0.0001).

(**B**) Expression of CAh7 in rectum of males at different time of day determined by RNA-seq (n = 5, *F*_(5,24)_ = 4.819, *P* = 0.0034).

(**C**) Expression of βCAh1 in rectum of males at different time of day determined by RNA-seq (n = 5, *F*_(5,24)_ = 10.91, *P* < 0.0001).

(**D**) Expression of CAh2-1 in rectum of males at different time of day determined by RNA-seq (n = 5, *F*_(5,24)_ = 6.319, *P* = 0.0007).

(**E**) Expression of CAh2 in rectum of males at different time of day determined by RNA-seq (n = 5, *F*_(5,24)_ = 13.12, *P* < 0.0001).

(**F**) Expression of CAh1 in rectum of males at different time of day determined by qPCR (n = 5, *F*_(5,24)_ = 10.34, *P* < 0.0001).

(**G**) Expression of CAh7 in rectum of males at different time of day determined by qPCR (n = 5, *F*_(5,24)_ = 2.325, *P* = 0.0741).

(**H**) Expression of βCAh1 in rectum of males at different time of day determined by qPCR (n = 5, *F*_(5,24)_ = 3.22, *P* = 0.023).

(**I**) Expression of CAh2-1 in rectum of males at different time of day determined by qPCR (n = 5, *F*_(5,24)_ = 8.663, *P* < 0.0001).

(**J**) Expression of CAh2 in rectum of males at different time of day determined by qPCR (n = 5, *F*_(5,24)_ = 17.68, *P* < 0.0001).

Different letters above the error bars indicate significant differences at the 0.05 level analyzed by ANOVA followed by Tukey’s test.

**
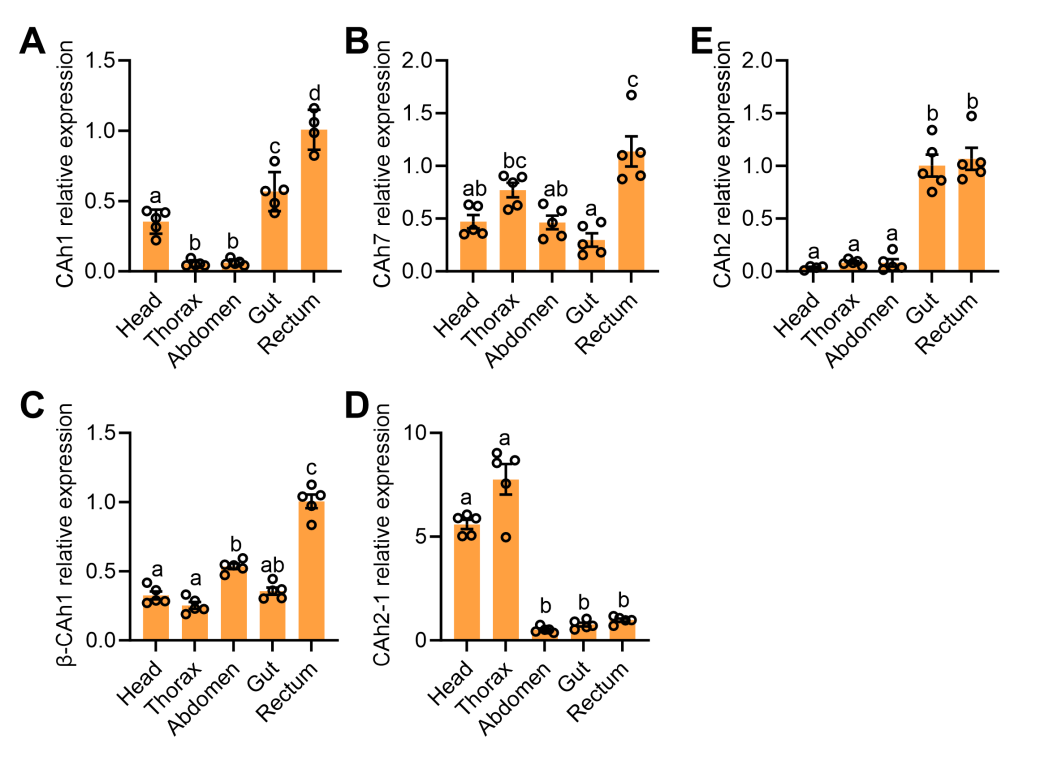
**

**Fig. S8. Expression of CAh genes in different tissues of mature male at 20:00.**

(**A**) Expression of CAh1 in different tissues of mature male at 20:00 (n = 4-5, *F*_(4,19)_ = 77.05, *P* < 0.0001).

(**B**) Expression of CAh7 in different tissues of mature male at 20:00 (n = 5, *F*_(4,20)_ = 14.69, *P* < 0.0001).

(**C**) Expression of βCAh1 in different tissues of mature male at 20:00 (n = 5, *F*_(4,20)_ = 95.19, *P* < 0.0001).

(**D**) Expression of CAh2-1 in different tissues of mature male at 20:00 (n = 5, *F*_(4,20)_ = 90.87, *P* < 0.0001, one-way ANOVA).

(**E**) Expression of CAh2 in different tissues of mature male at 20:00 (n = 5, *F*_(4,20)_ = 55.62, *P* < 0.0001).

Different letters above the error bars indicate significant differences at the 0.05 level analyzed by ANOVA followed by Tukey’s test. In the figures, gene expression levels were measured at 20:00.


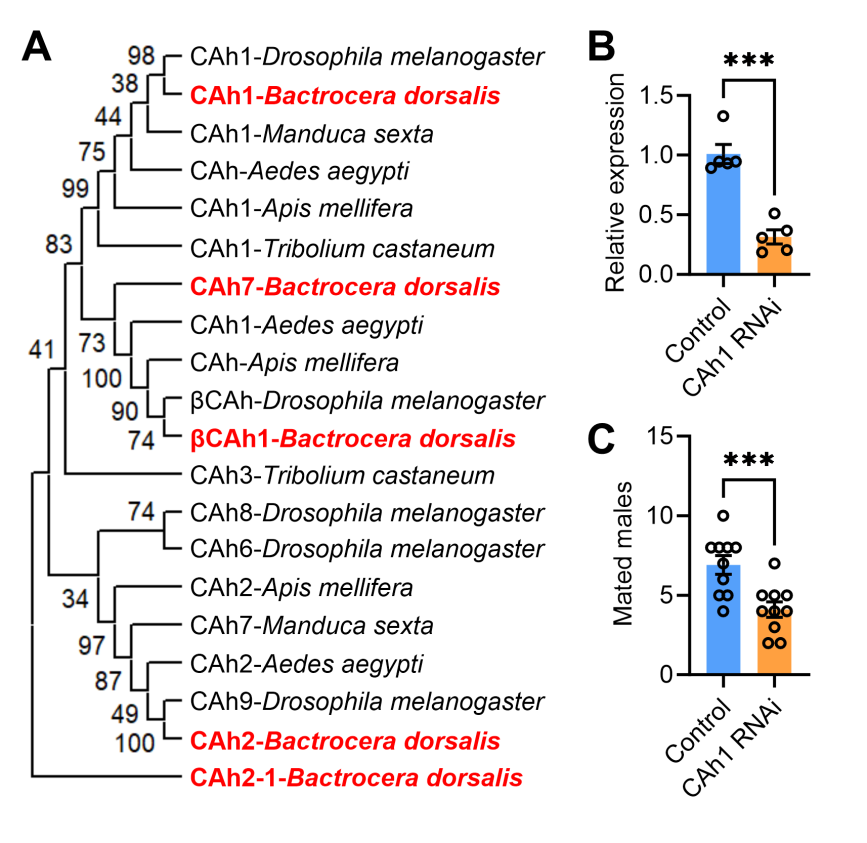


**Fig. S9. Highly expressed CAh1 is responsible for sex pheromone synthesis.**

(**A**) Maximum likelihood topology tree for diverse insect CAhs reveals conserved clades for CAhs of *B. dorsalis*. The accuracy of the tree was showed by the bootstrap values beside the branches by bootstrapping using 500 replicates.

(**B**) RNAi efficiency of CAh1 24 h after dsRNA injection (n = 5, *P* = 0.0001, independent sample Student’s *t* test).

(**C**) GC‒MS ion chromatograms of rectal extracts from males subjected to CAh1 knockdown and control males. To prevent the peaks of sex pheromones from different treatments from overlapping one another, the chromatograms were shifted.

(**D**) Influence of CAh1 knockdown on male mating ability (n = 10, *P* = 0.0004, paired sample Student’s *t* test).

In the figures, gene expression, sex pheromone levels and mating behavior were measured at 20:00.


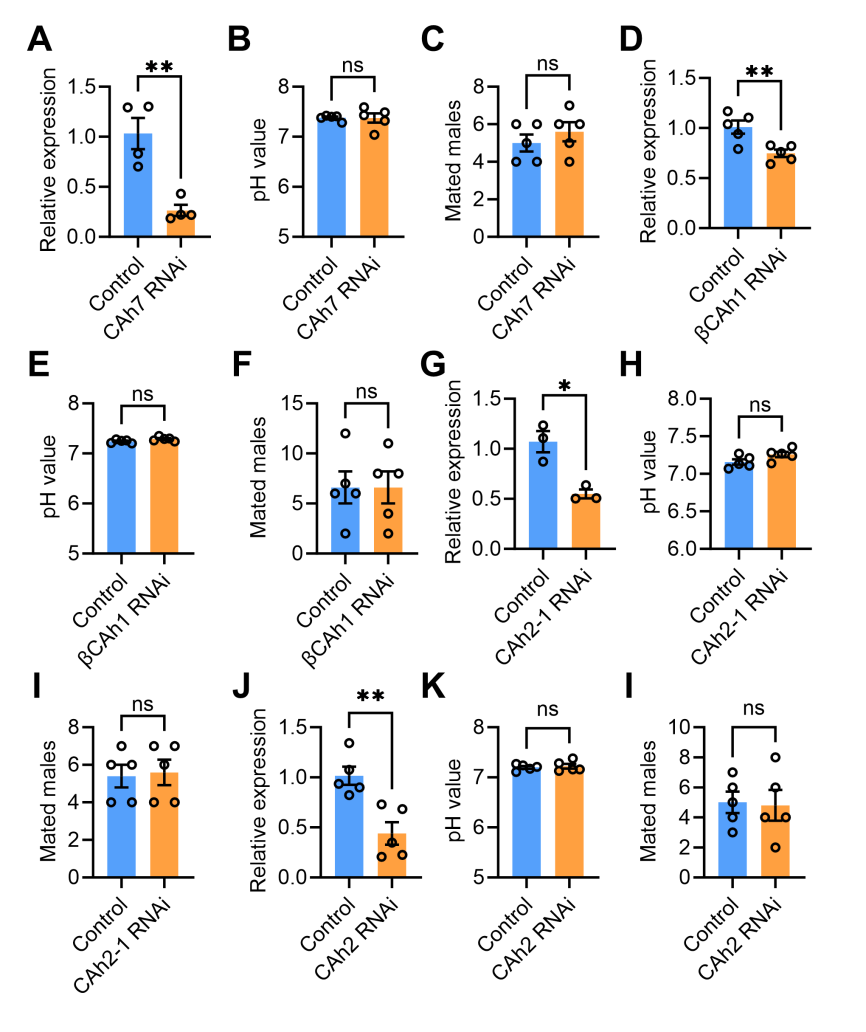


**Fig. S10. Knocking down expression of CAh7, βCAh1, CAh2-1 and CAh2 have no influence on male rectal pH and mating ability.**

(**A**) RNAi efficiency of CAh7 24h after dsRNA injection (n = 4, *P* = 0.0035, Independent sample student’s *t* test).

(**B**) Influence of CAh7 knocking down on rectum PH (n = 5, *P* = 0.9683, Independent sample student’s *t* test).

(**C**) Influence of CAh7 knocking down on male mating ability (n = 5, *P* = 0.208, Paired student’s *t* test).

(**D**) RNAi efficiency of βCAh1 24h after dsRNA injection (n = 5, *P* = 0.0087, Independent sample student’s *t* test).

(**E**) Influence of βCAh1 knocking down on rectum PH (n = 5, *P* = 0.0822, Independent sample student’s *t* test).

(**F**) Influence of βCAh1 knocking down on male mating ability (n = 5, *P* > 0.99, Paired student’s *t* test).

(**G**) RNAi efficiency of CAh2-1 24h after dsRNA injection (n = 3, *P* = 0.0104, Independent sample student’s *t* test).

(**H**) Influence of CAh2-1 knocking down on rectum PH (n = 5, *P* = 0.0839, Independent sample student’s *t* test).

(**I**) Influence of CAh2-1 knocking down on male mating ability (n = 5, *P* = 0.8307, Paired student’s *t* test).

(**J**) RNAi efficiency of CAh7 24h after dsRNA injection (n = 4, *P* = 0.004, Independent sample student’s *t* test).

(**K**) Influence of CAh7 knocking down on rectum PH (n = 5, *P* = 0.6986, Independent sample student’s *t* test).

(**L**) Influence of CAh7 knocking down on male mating ability (n = 5, *P* = 0.8712, Paired student’s *t* test).

In the figures, gene expression, pH and mating behavior were measured at 20:00.

**
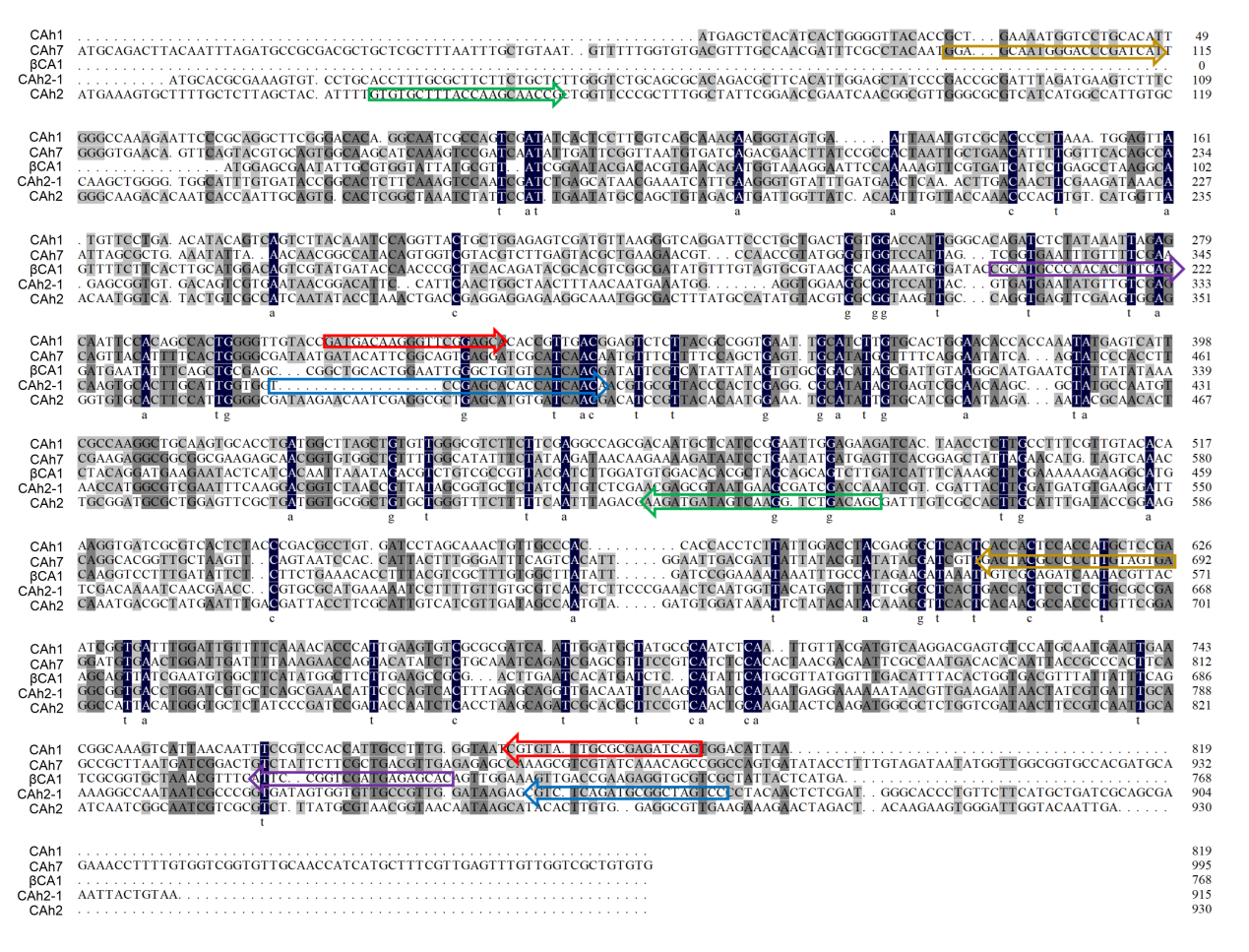
**

**Fig. S11. mRNA sequences alignments for all CAhs identified in male.** The colored arrows indicate the sites of primers for synthesizing dsRNA. Potential off-targets effect can be avoided, because the primer sites are very different from the sites of other CAs.

**Table S1 Primers used in the study**

|  | Gene | Primer name | sequence(5’-3’) | Product size |
| --- | --- | --- | --- | --- |
| Rectal bacteria 16S rRNA V3-V4 region amplification | 16S rRNA | 341F | CCTACGGGNGGCWGCAG | 466 |
|  |  | 806R | GGACTACHVGGGTATCTAAT |  |
| *Bacillus* abundance quantification | 16S rRNA | *Bacillus*-F | TGAAACTYAAAGGAATTGACG | 112 |
|  |  | *Bacillus*-R | ACCATGCACCACCTGTC |  |
| Amplification of TDH | TDH | F | AC**AAGCTT**CAATGTGCGGGCACCATGAAAGC | 1080 |
|  |  | R | TG**CTCGAG**TTTTTTGCCACGCTGGTAGCTCG |  |
| Primers used for gene quantification | CAh1 | CA1-F | TACGCCGGTGAATTGCATCT | 227 |
|  |  | CA1-R | GTGGGCAACAGTTTGCTAGG |  |
|  | βCAh1 | βCA1-F | GTCGTATGATACCAACCCGCT | 155 |
|  |  | βCA1-R | ATCGTTGATGACACAGCCCA |  |
|  | CAh2 | CA2-F | GGCCATTACATGGGTGCTCT | 152 |
|  |  | CA2-R | TACGCATAAAGACGCGACGA |  |
|  | CAh2-1 | CA3-F | TGCGTCAACTCTTCCCGAAA | 244 |
|  |  | CA3-R | ACGCTCTTATCCAACGGCAA |  |
|  | CAh7 | CA7-F | GGGGTGAACAGTTCAGTACGTGCAG | 205 |
|  |  | CA7-R | GGACCACCCCATACGGTTGG |  |
|  | RPL | RPL-F | CGATTTCTCCGCAGTATTCAC | 147 |
|  |  | RPL-R | GCCAGTACCTCATGCCTAACA |  |
|  | α-TUB | α-TUB-F | CGCATTCATGGTTGATAACG | 184 |
|  |  | α-TUB-R | GGGCACCAAGTTAGTCTGGA |  |
| Primers used for RNAi | CAh1 | CA1-F | GGATCCTAATACGACTCACTATAGGNGATGACAAGGGTTCGGAGCA | 503 |
|  |  | CA1-R | GGATCCTAATACGACTCACTATAGGNCTGATCTCGCGCAATACACG |  |
|  | βCAh1 | βCA1-F | GGATCCTAATACGACTCACTATAGGNGCATGCCCAACACTTTCAGG | 522 |
|  |  | βCA1-R | GGATCCTAATACGACTCACTATAGGNGTGCTCTCATCGACCGGAAT |  |
|  | CAh2 | CA2-F | GGATCCTAATACGACTCACTATAGGNGTGTGCTTTACCAAGCAACCGC | 524 |
|  |  | CA2-R | GGATCCTAATACGACTCACTATAGGNCGCTGTCAGACCTTGACTATCATCTT |  |
|  | CAh2-1 | CA3-F | GGATCCTAATACGACTCACTATAGGNTCCGAGCACACCATCAACAA | 503 |
|  |  | CA3-R | GGATCCTAATACGACTCACTATAGGNGGACTAGCCGCATCTGAGAC |  |
|  | CAh7 | CA7-F | GGATCCTAATACGACTCACTATAGGNGAGCAATGGGACCCGATCAT | 598 |
|  |  | CA7-R | GGATCCTAATACGACTCACTATAGGNTCACTACAAGGGGGCGTAGT |  |

Note: Restriction sites used in this study are shown in bold; The red sequence is the T7 sequence.
